# Heart failure etiology and lipoprotein subfractions: Insight from the SMARTEX-HF study

**DOI:** 10.1016/j.ijcha.2026.101888

**Published:** 2026-02-16

**Authors:** Trine Karlsen, Elisabeth K Vesterbekkmo, Torstein Hole, Alf Inge Larsen, Torstein Valborgland, Tonje Braaten, Tone F Bathen, Paul Beckers, Charles Delagardelle, Patrick Feiereisen, Emeline Van Craenenbroeck, Axel Linke, Eva Prescott, Martin Halle, Øyvind Ellingsen, Håvard Dalen

**Affiliations:** a Faculty of Nursing and Health Sciences, Nord University, Bodø, Norway; b Department of Circulation and Medical Imaging, NTNU – Norwegian University of Science and Technology, Trondheim, Norway; c Clinic of Cardiology, St. Olavs University Hospital, Trondheim, Norway; d Norwegian National Advisory Unit on Exercise Training as Medicine for Cardiopulmonary Conditions, St. Olavs University Hospital, Trondheim, Norway; e Ålesund Hospital, Møre og Romsdal Health Trust, Ålesund, Norway; f Department of Public Health and Nursing, NTNU – Norwegian University of Science and Technology, Trondheim, Norway; g Department of Cardiology, Stavanger University Hospital, Stavanger, Norway; h Department of Clinical Science, University of Bergen, Norway; i Department of Community Medicine, Faculty of Health Sciences, UiT The Arctic University of Norway, Norway; j Department of Rehabilitation Sciences and Physiotherapy, Faculty of Medicine and Health Sciences, University of Antwerp, Antwerp, Belgium; k Department of Cardiology, Centre Hospitalier de Luxembourg, Luxembourg; l Department of Cardiology, Antwerp University Hospital, Antwerp, Belgium; m Research group Cardiovascular Diseases, GENCOR, University of Antwerp, Antwerp, Belgium; n Heart Centre Dresden, University Hospital at Technical University of Dresden, Dresden, Germany; o Department of Cardiology, Bispebjerg Hospital, University of Copenhagen, Copenhagen, Denmark; p Department of Preventive Sports Medicine, TUM School of Medicine and Health, Technical University of Munich, Munich, Germany; q DZHK (German Centre for Cardiovascular Research), partner site Munich Heart Alliance, Munich, Germany; r Department of Medicine, Levanger Hospital, Nord-Trøndelag Hospital Trust, Levanger, Norway

**Keywords:** Heart failure, VLDL, IDL, HDL, LDL, Cardiomyopathy

## Abstract

**Background:**

We investigated the relationship between heart failure etiology and lipoprotein subfractions, and to explore their associations with left ventricular dimension and function in heart failure with reduced ejection fraction (HFrEF) patients.

**Methods:**

Cross-sectional investigation of serum lipoprotein subfractions from 205 HFrEF patients in the SMARTEX heart failure study. Serum levels of triglycerides, cholesterol, free cholesterol, phospholipids, lipoproteins (Apolipoproteins; A-1, A-2, and B), very-low-density (VLDL), intermediate-density (IDL), low-density (LDL), and high-density lipoprotein (HDL) were determined using ^1^H-Nuclear Magnetic Resonance spectroscopy.

**Results:**

Stable HFrEF patients [left ventricular ejection fraction (LVEF) ≤ 35%, NYHA class II-III], with ischemic (ICM, n = 119) or non-ischemic (NICM, n = 86) cardiomyopathy were studied. NICM patients had higher levels of 48 lipoproteins compared to ICM patients, including 29 LDL, 13 VLDL, and 6 HDL subfractions [p <0.05]. NICM patients had 22% higher cholesterol and 27% higher remnant cholesterol levels, with 24% more atherogenic ApoB containing subfractions (VLDL, IDL, LDL) (p <0.05). Heart failure etiology and statin treatment explained 23–24% of the variability in cholesterol, free cholesterol, and ApoB (p <0.001). Triglyceride content in some VLDL and LDL subfractions was weakly associated with left ventricular end-diastolic volume, end-diastolic diameter, ejection fraction, and S’.

**Conclusions:**

NICM patients had the highest atherosclerotic lipoprotein burden, attributed to elevated ApoB particles and partly due to less statin treatment. The triglyceride content of some VLDL and LDL subfractions was weakly associated with left ventricular structure and function. However, further research is needed to determine their prognostic significance before implementation into strategies for prevention and treatment.

**Trail Registration**: ClinicalTrial.gov database (NCT00917046)

## 1 Introduction

Hypercholesterolemia is a major risk factor for coronary artery disease (CAD)[1] and ischemic heart failure (HF).[2] Circulating lipoproteins are highly heterogeneous in structure, density, content, biological activity, and intravascular metabolism.[3], [4], [5], [6], [7] HF patients exhibit a unique lipoprotein profile[8], [9], [10], with the distribution of small high density lipoprotein (HDL) particles, and their cholesterol content emerging as promising prognostic biomarkers.[11], [12] A low number of small HDL particles with reduced cholesterol content has been linked to higher all-cause mortality,[9], [11], [12], [13] likely due to diminished anti-atherogenic, anti-inflammatory, and endothelial protective effects associated with HDL.[11], [12].

Compared to a matched control population from a large cohort study, HF-patients had larger atherogenic ApoB and anti-atherogenic ApoA rich lipoprotein particles. Additionally, both low-density lipoprotein (LDL) and HDL particles contained less cholesterol.[10] A low cholesterol content in very-low-density lipoprotein (VLDL) particles was previously associated with higher 1-year mortality in acute HF patients.[13] HF-etiology specific lipoprotein profiles have shown fewer LDL and HDL particles with lower triglycerides and cholesterol content in ischemic HF-patients compared to non-ischemic patients.[10] Thus, both atherogenic (VLDL and LDL) and anti-atherogenic (HDL) lipoprotein particles are linked to HF pathophysiology and prognosis, indicating a distinct HF-specific lipid biomarker profile.[9], [10], [11], [12], [13], [14], [15].

Lipoproteins may influence the myocardium independently of their pro- and anti-atherogenic effects on coronary artery atherosclerosis.[3] A recent UK biobank study suggested a causal relationship between LDL cholesterol and heart remodeling, with higher LDL cholesterol levels linked to increased left ventricular (LV) end-diastolic volume (LVEDV) and LV mass. Additionally, higher triglyceride levels were associated with increased LV-mass.[16] This indicates that lipoprotein composition might influence cardiac morphology beyond its role in atherosclerosis. Enhancing clinical knowledge of etiology-specific lipoprotein subfractions could improve the prevention and treatment of heart disease beyond traditional risk factors like LDL and HDL cholesterol.[17], [18] Therefore, our aim was to investigate HF etiology-specific lipoprotein subfractions and their association with left ventricular dimension and function in patients with stable ischemic and non-ischemic HFrEF.

## 2 Methods

This was a cross-sectional sub-study using baseline data from the *Study of Myocardial Recovery after Exercise Training in Heart Failure* (SMARTEX-HF) trial, including patients from 9 European study centers to 12 weeks of exercise training or control with 1-year follow-up.

### 2.1 Participants

The SMARTEX-HF study recruited and randomized 261 clinically stable HFrEF patients. Results and details of patient flow, inclusion and exclusion criteria have been comprehensively presented previously.[19], [20] In the current sub-study, 205 of the 215 patients completing the intervention had sufficient blood biobank samples to be included in the lipid sub-fraction analysis. At inclusion, all patients were on optimal HF treatment and had stable, symptomatic HFrEF with LVEF ≤ 35%, and NYHA class II-III. Etiology was ischemic (ICM) in n = 119 and non-ischemic (NICM) in n = 86.

The committees for medical research ethics approved the study for all recruiting centers, including written informed consent signed by all patients. The study was registered in the ClinicalTrial.gov database (NCT00917046) and conducted in conformity with the Declaration of Helsinki.

### 2.2 Clinical measurements

Medical history, anthropometrics, physical examination, fasting blood sampling, quality of life questionnaires, cardiopulmonary exercise testing (CPET), and echocardiography were performed at baseline.[20], [21].

Resting echocardiography data were acquired according to standard operation procedures of the study,[21] and images were analyzed at the core laboratory in Trondheim, Norway. Briefly, left ventricular (LV) volumes and LVEF were measured by biplane volumetric method in 4- and 2-chamber views. LV end-diastolic internal dimension (LVEDD) was measured at the level of the mitral tip in the parasternal long-axis. Peak mitral annular systolic velocity (S’) and peak mitral annular early diastolic velocity (e’) were measured in the 4-chamber view using pulsed-wave tissue Doppler.

### 2.3 Blood samples

Sixty ml serum, ethylenediaminetetraacetic acid (EDTA) and citrate plasma were collected at baseline and prepared for storage in the study biobank. Blood was sampled from an antecubital vein, in the morning, after 10 min of supine rest and overnight fasting. Vacutainers with blood and plasma were stored in ice water for 30 min before 10 min of centrifugation at 4°C and 1500 g. Serum and plasma were aliquoted in 500 μl aliquots and immediately frozen at −80°C. Samples were stored in the study biobank until the serum samples were used for lipid profiling.

Biochemical analyses of hematology, serum creatinine, triglycerides, total cholesterol, HDL, and LDL cholesterol were performed consecutively at each local hospital. N-terminal pro-Brain Natriuretic Peptide (NT-proBNP) and high-sensitive C-reactive protein (Hs-CRP) were analyzed at the Department of Clinical Chemistry at St. Olavs University Hospital, Trondheim, Norway (Table 1).

**Table 1:** Patients baseline characteristics.

Characteristics	Total (n = 205)	ICM (n = 119)	NICM (n = 86)
Women, n (%)	40 (19.5)	17 (14.3) *	23 (26.7)
Age (years)	61.1 ± 11.8	63. ± 11.1*	58.2 ± 12.2
Height (cm)	174.8 ± 8.8	175.5 ± 8.2	173.8 ± 9.5
Body mass index (kg·m^2^)	28.2 ± 5.0	28.1 ± 4.9	28.4 ± 5.1
Heart failure < 12 months, n (%)	36 (17.6)	17 (14.3)	19 (22.1)
LVEDD (mm)	68.3 ± 8.0	68.5 ± 8.2	68.1 ± 7.8
LVEDV (mL)	249.7 ± 75.5	241.4 ± 37.7	261.2 ± 84.2
S’ (cm/s)	5.0 ± 1.4	4.9 ± 1.3	5.2 ± 1.5
e’ (cm/s)	6.0 ± 2.0	6.2 ± 1.9	5.9 ± 1.9
E/e’	13.5 ± 7.7	13.8 ± 8.0	13.2 ± 7.3
LVEF (%)	28.7 ± 6.6	28.6 ± 6.1	28.9 ± 7.3
NYHA II	144 (70.2)	80 (67.2)	64 (74.4)
NYHA III	61 (29.8)	39 (32.8)	22 (25.6)
Systolic blood pressure (mmHg)	120 ± 17	119 ± 16	120 ± 19
Diastolic blood pressure (mmHg)	74 ± 11	73 ± 10	75 ± 12
VO_2peak_ (L∙min^−1^)	1.71 ± 0.57	1.64 ± 0.53*	1.81 ± 0.62
VO_2peak_ (ml∙kg^−1^∙min^−1^)	17.7 ± 4.9	16.8 ± 4.4*	18.9 ± 5.2
NT-proBNP(ng/L)	979.0 (421.0–1824.0)	1000.0 (542.0–1847.0)	975.5 (343.5–1831.8)
S-Creatinine (µmol/l)	92.4 (80.0–109.6)	95.2 (82.8–121.3)*	89.3 (76.5–107.3)
GRF (ml/min)	86.6 (62.3–107.0)	86.3 (57.2–107.0)	87.6 (66.6–108.2)
Hs-CRP (mg/L)	1.89 (0.88–4.00)	1.89 (0.85–3.82)	1.91 (0.90–4.12)
Hb (g/dl)	14.2 (13.2–15.2)	14.2 (13.0–15.2)	14.2 (13.2–15.3)
Triglycerides (mmol/L)	1.50 (1.00–2.20)	1.35 (0.90–2.10)*	1.60 (1.00–2.30)
Total cholesterol (mmol/L)	4.50 (3.80–5.70)	4.10 (3.60–5.00)*	5.40 (4.60–6.30)
HDL cholesterol (mmol/L)	1.10 (0.90–1.40)	1.10 (0.90–1.40)*	1.20 (1.00–1.50)
LDL cholesterol (mmol/L)	2.80 (2.02–3.56)	2.40 (1.90–3.00)*	3.40 (2.80–4.15)
Previous MI, n (%)	106 (52.7)	103 (86.6)*	3 (3.5)
History of CABG, n (%)	49 (23.9)	49 (41.2)*	0 (0.0)
History of PCI, n (%)	82 (40.0)	80 (67.2)*	2 (2.3)
History of hypertension, n (%)	77 (37.6)	41 (34.5)	36 (41.9)
History of diabetes mellitus, n (%)	46 (22.4)	31 (26.1)	15 (17.4)
Current smoker, n	37 (18.0)	21 (17.6)	16 (18.6)
Alcohol (drinks·week^−1^)	3.8 ± 5.4	3.6 ± 4.7	4.1 ± 6.3
Device therapy, n (%)			
Pacemaker	4 (2.0)	2 (1.7)	2 (2.3)
Implantable cardioverter defibrillator	90 (43.9)	59 (49.6)	31 (36.0)
Cardiac resynchronization therapy	28 (13.6)	17 (14.3)	11 (12.8)
Atrial fibrillation, n (%)			
Paroxysmal	25 (12.2)	14 (11.8)	11 (12.8)
Persistent	28 (13.7)	16 (13.4)	12 (14.0)
Medications, n (%)			
Anticoagulation, n (%)	72 (35.1)	39 (32.8)	33 (38.4)
ACE inhibitor, n (%)	137 (66.8)	79 (66.4)	58 (67.4)
Betablockers, n (%)	196 (95.6)	112 (94.1)	84 (97.7)
Diuretics, n (%)	148 (72.2)	87 (73.1)	61 (70.9)
Digoxin or digitoxin, n (%)	29 (14.1)	15 (12.6)	14 (16.3)
Statins, n (%)	134 (65.4)	110 (92.4)*	24 (27.9)
ARB, n (%)	60 (29.3)	38 (31.9)	22 (25.6)
Acetylsalicylic acid, n (%)	113 (55.1)	93 (78.2)*	20 (23.3)
Clopidogrel, n (%)	25 (12.2)	23 (19.3)	2 (2.3)
Cordarone (amiodarone), n (%)	23 (11.2)	13 (10.9)	10 (11.6)
Oral antidiabetic, n (%)	29 (14.1)	19 (16.0)	10 (11.6)
Insulin, n (%)	21 (10.2)	15 (12.6)	6 (7.0)
Spironolacton/eplerenone, n (%)	116 (56.6)	65 (54.6)	51 (59.3)

Data is displayed in means and standard deviations, medians with interquartile range, or as frequencies with percentages. ICM = Ischemic cardiomyopathy group; NICM = Non-ischemic cardiomyopathy group; S’ = Mitral annular systolic peak velocity; e’ = mitral annular diastolic velocity; LVEDD = left ventricular end-diastolic diameter; LVEDV = Left ventricular end-diastolic volume; LVEF = Left ventricular ejection fraction; NYHA = New York heart association classification; VO_2peak_ = peak oxygen uptake; NT-proBNP = N-terminal pro-brain natriuretic peptide; S = serum; GRF = Estimated glomerular filtration rate; Hs-CRP = high sensitive C-reactive protein; Hb = Hemoglobin; HDL = high-density lipoprotein; LDL = low-density lipoprotein; MI = myocardial infarction; CABG = coronary artery bypass graft; PCI = percutaneous coronary intervention; ACE = angiotensin-converting-enzyme inhibitor; ARB = angiotensin II receptor blockers. * Indicates significant differences between the ICM and NICH groups (p ≤ 0.05).

### 2.4 Lipoprotein subfraction analysis

Serum blood samples were analyzed for lipoprotein subfractions by ^1^H-Nuclear Magnetic Resonance (^1^H NMR) spectroscopy at the MR Core Facility at the Norwegian University of Science and Technology, NTNU, in Trondheim, Norway. The Bruker Avance III 600 MHz spectrometer (Bruker BioSpin, GmBH, Rheinstetten, Germany) with SampleJet autosampler and a 5 mm QCI Cryoprobe for 1H/ [31P, 13C, 15 N] was used. 1D-1H Nuclear Overhauser effect spectroscopy (NOESY) and Carr-Purcell-Meiboom-Gill (CPMG) spectra with water pre-saturation were obtained at 310 K. The spectra were Fourier-transformed to 128 K data points after 0.3 Hz exponential line broadening. An automated Bruker IVDr Lipoprotein Subclass Analysis (B.I.LISA^TM^) was used to quantify lipoprotein variables [22]. Different composition, size, and density of lipoproteins gives different NMR signal shapes that transfers into information about lipoprotein fractions and subfractions.

In preparation for analysis, serum samples were slowly thawed overnight in a refrigerator at 4°C and aliquots of 150 μl serum were mixed with 150 μl buffer (20% D_2_O in H_2_O with 0.075 M Na_2_HPO_4_, 6 mM NaN_3_, 4.6 mM trimethylsilylpropanoic acid (TSP), pH 7.4) and the solution transferred to 3 mm NMR tubes.

The total serum concentrations of triglycerides, cholesterol, free cholesterol, phospholipids, and the apolipoproteins A-1, A-2, and B were determined. In each of the four lipoproteins, VLDL, intermediate-density lipoprotein (IDL), LDL, and HDL, the content of triglycerides, cholesterol, free cholesterol, and phospholipids was determined. The content of ApoA-1 and ApoA-2 was determined in serum HDL particles, and the content of ApoB was determined in serum VLDL, IDL, and LDL particles, respectively.

**Fig. 1:**
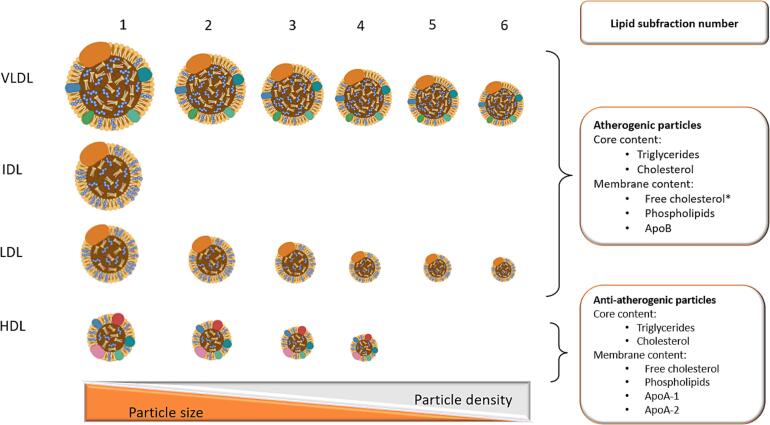
**Overview of lipoprotein fractions and subfractions measured by ^1^H NMR** Illustration of particle sizes of very-low-density lipoproteins (VLDL), intermediate-density lipoproteins (IDL), low-density lipoproteins (LDL), and high-density lipoprotein (HDL) fractions, and their subfractions (VLDL 1–6, LDL 1–6, HDL 1–4). Core content of triglycerides and cholesterol, and membrane content of free-cholesterol, phospholipids and apolipoprotein-B (Apo-B) were measured in the atherogenic VLDL, IDL and LDL fractions and subfractions (*free cholesterol was not measured in IDL particles). Core content of triglycerides and cholesterol, and membrane content of free-cholesterol, phospholipids and apolipoprotein-A1(Apo-A1) and Apo-A2 were measured in the non-atherogenic HDL fractions and subfractions.

The content of triglycerides, cholesterol, free cholesterol, and phospholipids was further measured in the 16 lipoprotein subfractions; VLDL 1 to 6, LDL 1 to 6, and HDL 1 to 4, defined by size and density. The VLDL and LDL subfractions were differentiated by size from 1 (the largest and least dense particles) to 6 (the smallest and most dense particles). HDL 1 was the largest and least dense HDL subfraction with HDL 4 the smallest and most dense. ApoB was determined in LDL 1 to 6, and ApoA-1 and ApoA-2 were determined in the HDL 1 to 4. In total, this yielded 78 lipoprotein subfraction variables for VLDL, LDL, and HDL with increasing particle number corresponding to decreasing particle size and increasing particle density, as well as the IDL fraction where no subfractions were measured (Fig. 1, Table 2, Table 3, Table 4, Table 5).

**Table 2:** Lipoprotein fractions by heart failure etiology.

ICM (n = 119)	Cholesterol	Triglycerides	Free cholesterol	Phospholipids	Apo-A1	Apo-A2	Apo-B
Serum (mg/dl)	139.7 (133.5–144.9)	117.1 (106.2–130.6)	49.8 (48.2–52.0)	−	118.9 (115.1–123.3)	23.5 (22.8–24.6)	65.9 (63.6–71.3)
VLDL (mg/dl)	19.6 (16.9–21.0)	71.8 (61.5–80.1)	10.0 (9.1–11.2)	19.7 (18.1–22.6)	−	−	9.1 (8.3–9.6)
IDL (mg/dl)	6.4 (5.6–7.4)	8.6 (6.5–10.2)	1.6 (1.3–1.9)	3.1 (1.9–3.5)	−	−	3.1 (2.7–3.6)
LDL (mg/dl)	63.5 (57.8–70.0)	17.1 (15.7–18.2)	22.6 (21.3–23.6)	40.6 (37.3–42.3)	−	−	49.7 (45.8–51.8)
HDL (mg/dl)	43.0 (41.4–45.0)	9.4 (8.7–9.8)	13.2 (12.7–13.8)	58.6 (56.0–61.4)	119.2 (116.0–125.0)	24.9 (24.2–26.1)	−
NICM (n = 86)							
Serum (mg/dl)	179.3 (173.2–195.5)*	134.8 (125.0–152.5)*	61.1 (57.8–66.0)*	−	127.5 (122.2–130.7)*	25.5 (24.6–26.5)*	86.8 (79.8–93.1)*
VLDL (mg/dl)	25.1 (20.5–28.3)*	87.3 (72.9–96.4)	11.8 (10.8–13.3)*	24.1 (21.1–26.2)	−	−	10.8 (9.4–11.7)*
IDL (mg/dl)	10.5 (8.5–13.5)*	11.8 (8.7–13.9)*	2.8 (2.3–3.6)*	4.6 (3.8–6.0)*	−	−	4.4 (3.9–5.4)*
LDL (mg/dl)	92.6 (80.4–99.5)*	20.0 (18.5–21.9)*	30.0 (26.5–31.6)*	54.3 (47.7–57.3)*	−	−	67.3 (62.5–70.8)*
HDL (mg/dl)	46.0 (43.3–48.8)	9.4 (8.7–10.3)	13.7 (13.1–15.4)	63.4 (60.1–65.5)	127.1 (121.6–133.1)	26.7 (25.8–27.7)*	−

Data is displayed in median with 95% interquartile range. APO = apolipoprotein; ICM = ischemic cardiomyopathy group; VLDL = very-low-density lipoprotein fraction; IDL = intermediate-density lipoprotein fraction; LDL = low-density lipoprotein fraction; HDL = high-density lipoprotein fraction; NICM = non-ischemic cardiomyopathy group. *Indicates significant differences between the ICM and NICH groups (p ≤ 0.05).

**Table 3:** Lipoprotein content in VLDL subfractions by heart failure etiology.

ICM (n = 119)	Cholesterol	Triglycerides	Free cholesterol	Phospholipids
VLDL 1 (mg/dl)	6.7 (6.0–7.9)	33.4 (27.9–38.8)	1.3 (0.9–1.8)	4.4 (3.7–5.5)
VLDL 2 (mg/dl)	2.9 (2.3–3.1)	10.9 (9.4–13.3)	1.2 (1.0–1.5)	2.4 (2.0–3.0)
VLDL 3 (mg/dl)	2.4 (2.1–2.8)	10.5 (8.8–11.1)	1.2 (1.1–1.5)	2.8 (2.3–3.1)
VLDL 4 (mg/dl)	3.9 (3.4–4.5)	9.1 (8.0–9.8)	1.6 (1.4–1.8)	4.3 (3.9–4.6)
VLDL 5 (mg/dl)	2.0 (1.9–2.0)	4.1 (3.9–4.2)	1.2 (1.1–1.3)	2.5 (2.4–2.6)
VLDL 6 (mg/dl)	0.00 (0.00–0.00)	0.69 (0.44–0.83)	0.096 (0.095–0.100)	0.245 (0.238–0.249)
NICM (n = 86)				
VLDL 1 (mg/dl)	9.0 (6.9–10.5)*	39.5 (33.9–48.4)	1.9 (1.4–2.5)	5.5 (4.5–6.9)
VLDL 2 (mg/dl)	3.7 (3.0–4.3)*	13.2 (10.6–15.2)	1.7 (1.3–1.9)*	3.0 (2.3–3.6)
VLDL 3 (mg/dl)	3.6 (2.4–4.1)*	11.9 (10.0–12.8)	1.7 (1.2–2.0)*	3.5 (2.5–3.9)
VLDL 4 (mg/dl)	5.4 (4.4–5.9)*	10.2 (8.7–12.1)	2.1 (1.8–2.5)*	5.3 (4.5–5.7)*
VLDL 5 (mg/dl)	1.9 (1.8–2.2)	4.2 (3.9–4.5)	1.4 (1.2–1.5)*	2.6 (2.4–2.7)
VLDL 6 (mg/dl)	0.00 (0.00–0.00)	1.01 (0.91–1.10)*	0.101(0.098–0.102)*	0.259 (0.250–0.261)*

Data is displayed in median with 95% interquartile range. Apo = apolipoprotein; ICM = ischemic cardiomyopathy group; NICM = non-ischemic cardiomyopathy group; VLDL = very-low-density lipoprotein. *Indicates significant differences between the ICM and NICH groups (p ≤ 0.05).

**Table 4:** Lipoprotein content in LDL subfractions by heart failure etiology.

ICM (n = 119)	Cholesterol	Triglycerides	Free cholesterol	Phospholipids	Apo-B
LDL 1 (mg/dl)	12.5 (11.6–13.5)	5.7 (5.3–6.1)	3.8 (3.6–4.1)	8.1 (7.8–8.8)	8.3 (7.5–8.8)
LDL 2 (mg/dl)	7.1 (6.6–8.3)	1.5 (1.4–1.6)	3.2 (2.9–3.4)	4.7 (4.3–5.4)	5.2 (4.8–5.6)
LDL 3 (mg/dl)	5.2 (4.0–6.3)	2.5 (2.4–2.6)	3.5 (3.1–3.8)	3.9 (3.2–4.4)	4.1 (3.6–4.7)
LDL 4 (mg/dl)	7.6 (6.2–9.2)	2.0 (1.8–2.2)	3.0 (2.7–3.5)	4.8 (4.1–5.5)	5.5 (4.3–6.5)
LDL 5 (mg/dl)	11.6 (10.1–13.9)	2.4 (2.2–2.6)	4.0 (3.3–4.1)	6.7 (6.2–7.9)	9.1 (7.9–9.9)
LDL 6 (mg/dl)	20.5 (18.0–22.0)	4.3 (4.0–4.6)	4.6 (4.3–5.0)	11.3 (10.5–12.3)	17.7 (16.0–19.1)
NICM (n = 86)					
LDL 1 (mg/dl)	18.3 (15.3–20.2)*	6.8 (6.4–7.8)*	5.7 (4.8–6.2)*	10.7 (9.9–11.9)*	10.6 (10.0–11.6)*
LDL 2 (mg/dl)	11.1 (9.1–12.8)*	2.0 (1.8–2.3)*	4.2 (3.8–4.7)*	6.7 (5.6–7.5)*	6.9 (6.3–8.1)*
LDL 3 (mg/dl)	9.0 (7.5–10.7)*	2.8 (2.6–3.1)*	4.3 (3.9–5.0)*	5.7 (5.0–6.5)*	6.1 (5.4–6.6)*
LDL 4 (mg/dl)	11.8 (9.9–13.7)*	2.3 (2.2–2.6)*	4.2 (3.6–4.5)*	6.9 (6.0–7.9)*	8.0 (7.0–9.1)*
LDL 5 (mg/dl)	15.2 (11.6–16.3)*	2.8 (2.4–3.3)*	4.5 (3.8–4.9)*	8.3 (6.7–9.1)*	10.5 (9.0–12.0)*
LDL 6 (mg/dl)	23.7 (21.6–26.8)*	4.8 (4.3–5.3)	5.4 (4.5–6.1)*	13.1 (11.9–14.8)*	21.3 (19.1–23.6)*

Data is displayed in median with 95% interquartile range. Apo = apolipoprotein; ICM = ischemic cardiomyopathy group; LDL = low-density lipoprotein; NICM = non-ischemic cardiomyopathy group. *Indicates significant differences between the ICM and NICH groups (p ≤ 0.05).

**Table 5:** Lipoprotein content in HDL subfractions by heart failure etiology.

ICM (n = 119)	Cholesterol	Triglycerides	Free cholesterol	Phospholipids	Apo-A1	Apo-A2
HDL 1 (mg/dl)	9.4 (8.4–10.7)	2.3 (2.1–2.5)	2.7 (2.4–2.9)	10.8 (9.9–12.1)	13.5 (12.0–15.9)	0.97 (0.71–1.32)
HDL 2 (mg/dl)	5.2 (4.9–6.0)	1.4 (1.3–1.5)	1.2 (1.1–1.4)	8.6 (7.8–9.3)	14.0 (13.0–14.8)	1.5 (1.4–1.8)
HDL 3 (mg/dl)	7.5 (7.3–8.1)	1.9 (1.8–2.0)	1.6 (1.4–1.7)	12.0 (11.3–12.8)	22.0 (21.2–23.2)	4.0 (3.7–4.4)
HDL 4 (mg/dl)	18.3 (17.4–19.6)	3.8 (3.5–3.9)	3.7 (3.5–3.9)	24.3 (23.5–26.5)	71.2 (68.7–74.2)	17.1 (16.0–18.0)
NICM (n = 86)						
HDL 1 (mg/dl)	11.4 (9.1–12.2)*	2.4 (1.9–2.8)	3.2 (2.7–3.5)	12.7 (10.9–14.1)	15.6 (13.6–18.2)	1.4 (1.2–1.8)*
HDL 2 (mg/dl)	5.9 (5.5–6.4)	1.4 (1.2–1.5)	1.4 (1.3–1.5)	9.5 (8.9–9.8)	14.7 (13.8–15.8)	2.0 (1.8–2.2)*
HDL 3 (mg/dl)	8.6 (7.9–9.1)*	2.0 (1.8–2.2)	1.8 (1.7–2.0)*	13.5 (12.1–14.1)	25.0 (23.2–26.1)*	4.7 (4.3–5.0)*
HDL 4 (mg/dl)	19.5 (18.2–20.5)	3.9 (3.7–4.2)	4.0 (3.7–4.4)	26.2 (25.0–27.3)	73.8 (70.2–77.1)	17.9 (17.1–18.6)

Data is displayed in median with 95% interquartile range. Apo = apolipoprotein; HDL = high-density lipoprotein; ICM = ischemic cardiomyopathy group; NICM = non-ischemic cardiomyopathy group. *Indicates significant differences between ICM and NICH groups (p ≤ 0.05).

### 2.5 Statistical analysis

Demographic data are presented as means and standard deviations, median with interquartile range, or as frequencies with percentages. Independent sample *t*-tests were used to evaluate demographic differences between ICM and NICM patients. Lipid subfractions are presented as median with a 95% interquartile range. The non-parametric independent sample Mann-Whitney U Test was used to evaluate differences in lipoproteins between ICM and NICM patients.

Simple and multiple linear regression analysis were performed to investigate how HF etiology, statin treatment, age, BMI, or VO_2peak_ influenced lipid fractions and subfractions. Pearson correlation analysis was used to evaluate the correlation between standard biochemical measured lipids and ^1^H NMR spectroscopy.

The associations of lipid subfractions with LV size (LVEDV and LVEDD), and LV function (S’, e’, E/e’ ratio and LVEF) were tested using mixed-effect multilevel regression and variable selection with least absolute shrinkage and selection operator (LASSO). The LV models were adjusted for age, sex, BMI, and HF etiology.

Statistical analyses were performed with IBM SPSS statistics (version 23) or STATA for Windows, version 17.0, StataCorp LLC, 2017. A two-sided p ≤ 0.05 was defined as statistically significant.

## 3 Results

### 3.1 Demographics

Notable differences between the groups included a lower proportion of women, older age, lower fitness levels, and a higher prevalence of previous myocardial infarction (MI), percutaneous coronary intervention (PCI), and coronary artery bypass grafting (CABG), as well as more frequent use of statins and acetylsalicylic acid in the ICM group.

### 3.2 Biomarkers

Biochemical determined triglyceride, total cholesterol, LDL, and HDL cholesterol levels were 26%, 21%, 27%, and 10% higher in the NICM group compared to the ICM group, respectively. Serum creatinine was also higher in the NICM group (p ≤ 0.05). All other demographic variables were similar across groups (Table 1). ^1^H NMR-measured triglyceride, total cholesterol, HDL- and LDL cholesterol were significantly correlated with standard biochemical measurements; triglyceride (r = 0.963), total cholesterol (r = 0,876), HDL cholesterol (r = 0.773), and LDL cholesterol (r = 0.844) (p < 0.001).

### 3.3 ^1^H NMR-measured lipoprotein fractions and subfractions

Serum from NICM patients contained 13–22% more triglycerides, cholesterol, and free cholesterol than ICM patients. Remnant cholesterol (i.e., VLDL and IDL cholesterol) was 27% higher in the NICM group (p < 0.05, Table 2). Of the 78 lipoprotein subfractions analyzed, 48 (61.5%) were higher in the NICM group, including 29 LDL, 13 VLDL, and 6 HDL subfractions (p < 0.05, Table 3, Table 4, Table 5).

ApoA-1, ApoA-2, and ApoB levels were 7%, 8%, and 24% higher in the NICM group (p ≤ 0.024, Table 2). Since VLDL, LDL, and IDL particles each contain one ApoB molecule, NICM patients had 24% more circulating atherogenic particles.

HF etiology (ICM or NICM) and statin therapy (yes or no) explained some of the variations in total triglycerides, ApoA-1, and ApoA-2 (p ≤ 0.22, Supplementary Tables 1 and 2). These factors account for 23–24% of the variability in total cholesterol, free cholesterol, and ApoB particles. (R^2^ 0.23–0.24, p <0.001). Adding variables such as age, BMI or VO_2peak_ to the regression model had a minimal impact on these associations (Supplementary Tables S3–5).

### 3.4 Very-low-density lipoproteins

VLDLs were the primary carriers of triglycerides, containing 61–65% of total triglycerides. The VLDL triglyceride content was similar in both groups (Table 2), with a VLDL-to-triglyceride ratio (VLDL triglyceride/ApoB) of 8.1 in the ICM and 7.9 in the NICM group. In both groups, VLDL 1 contained ∼49% of the total triglycerides distributed across the VLDL1-6 subfractions. VLDL ApoB levels were 16% higher in the NICM group than in the ICM group, indicating more atherogenic VLDL particles in the NICM group (p ≤ 0.05, Table 2). Additionally, the NICM group exhibited 22% more VLDL cholesterol, with significantly elevated cholesterol in the VLDL 1–4 subfractions compared to the ICM group (all p ≤ 0.05, Table 3).

HF etiology alone, or in combination with statin treatment, explained ≤ 5.0% of the variability in VLDL fractions, and up to ∼9% of the variability in lipid and protein contents in VLDL 1–6 subfractions (p <0.001to p = 0.942), Supplementary Tables 1 and 2).

### 3.5 Intermediate-density lipoproteins

IDL ApoB levels were 30% higher in the NICM group, indicating more atherogenic IDL particles compared to the ICM group. The IDL content of cholesterol and triglycerides was 27–39% higher in the NICM group (Table 2, Fig. 1, p ≤ 0.05). The cholesterol content per IDL fraction (IDL cholesterol/ApoB) was 2.1 in the ICM group and 2.4 in the NICM groups.

HF etiology and statin treatment explained < 5% of the variability in IDL triglyceride and phospholipid content (p <0.065), and ∼9–12% of the variability in IDL cholesterol, free cholesterol, and ApoB content (p <0.001, Supplementary Tables 1 and 2).

### 3.6 Low-density lipoproteins

LDL ApoB levels were 26% higher in the NICM group, indicating more atherogenic LDL particles compared to the ICM group. Additionally, LDL cholesterol, the major component of LDL fractions, was 31% higher in the NICM group (p ≤ 0.05, Table 2). The cholesterol content per LDL fraction (LDL cholesterol/ApoB) was 1.3 in the ICM and 1.4 in the NICM groups. LDL triglyceride was 31% higher in the NICM group (p ≤ 0.05, Table 2).

Both groups had approximately twice as many small dense LDL 6 subfractions as the large and least dense LDL 1 subfractions. LDL 1–5 triglyceride content was 11–25% higher, LDL 1–6 ApoB content was 13–33% higher, and LDL 1–6 cholesterol 14–42% higher in the NICM group (p ≤ 0.05, Table 4).

HF etiology and statin therapy explained 12–27% of the variability in LDL content of triglyceride, cholesterol, free cholesterol, phospholipid, and ApoB, and between 2–24% of the variability in LDL 1–6 cholesterol, triglyceride, ApoB, free cholesterol and phospholipid content (p <0.005 to p <0.124, Supplementary Tables 1 and 2). A sub-analysis of all patients receiving statin therapy showed comparable LDL cholesterol levels in the ICM and NICM groups, suggesting similar adherence to prescribed statin therapy.

### 3.7 High-density lipoproteins

Apart from a 5% higher ApoA-2 content in the NICM group (p ≤ 0.05), HDL fractions were equal between groups (Table 2). The ApoB/ApoA-1 ratio was 17% higher in NICM group (0.70 ± 0.19) compared to the ICM group (0.60 ± 0.16), with a statistically significant difference (p < 0.001). In HDL 1 and HDL 3 subfractions, the cholesterol content was 13–18% higher in the NICM group (p ≤ 0.05, Table 5). Among all HDL subfractions, HDL 4 contained most cholesterol, phospholipid, ApoA-1, and ApoA-2, with the highest levels observed in the NICM group (p ≤ 0.05, Table 5).

### 3.8 Associations with echocardiographic measures of LV size and function

The LASSO regression analyses adjusted for age, sex, BMI, and HF etiology revealed significant associations with LVEDV for triglyceride content of the VLDL 5 subfraction (p = 0.032) and LDL 2 subfraction (p = 0.011) (Supplementary Table S6). Similarly, the triglyceride content of the VLDL 6 (p = 0.032), and LDL 2 (p = 0.064) subfractions were weakly associated with LVEDD. Beyond the associations of the triglyceride content of VLDL 5 with S’, none of the other VLDL subfractions were significantly associated with LVEF or S’ (Supplementary Table S6). There was no between the group interactions affecting the analyses.

## 4 Discussion

Our main finding was: 1) Patients with non-ischemic etiology had a higher atherogenic burden, with more circulating ApoB carrying particles, compared to patients with ischemic etiology. This included both triglyceride-rich VLDL and IDL remnant cholesterol fractions, as well as LDL fractions. 2) Approximately 76–78% of the ApoB carrying subfractions were LDLs, while VLDLs and IDLs together accounted for ∼18–19% of the ApoB containing particles. 3) HF etiology and statin therapy collectively accounted for up to 24% of the variability in lipid fraction content of cholesterol and ApoB. 4) LV size, but not function, was weakly associated with triglyceride content in certain VLDL and LDL subfractions.

### 4.1 Apo lipoprotein B and Apo lipoprotein A

Despite a non-ischemic HF origin, NICM patients exhibited a higher atherogenic lipid burden, with more ApoB containing atherogenic VLDL, IDL, and LDL particles compared to ICM patients. However, the ApoB content was below the suggested coronary heart disease risk cut-off of ≥ 1.20 g⋅L^−1^ in both etiology groups.[24] The amount of ApoB containing LDL fractions was comparable to the 80–90% LDL ApoB fractions reported in other studies.[24], [25], [26], [27] Less ApoB particles in the ICM group were not fully explained by statin treatment, likely because statins are more effective at lowering LDL cholesterol than reducing the content of ApoB containing particles.[26] The ApoB/ApoA-I ratio, reflecting atherogenic versus anti-atherogenic properties, was highest in the NICM group (0.70), a value that is borderline to high cholesterol-associated risk of cardiovascular disease.[26] The ApoB/ApoA-I ratio was 0.60 in our ICM patients, comparable to that in CAD patients in a previous study.[25] However, the prognostic value of both ApoB and the ApoB/ApoA-1 relationship requires further investigation in HF populations.

### 4.2 Very-low-density lipoproteins

As in one previous study,[10] we found equal triglyceride content in VLDL fractions and VLDL 1–5 subfractions in both HFrEF etiologies. The triglyceride ratio per VLDL particle was ∼8 in our patients, and comparable to ratios of ∼6 and ∼10 reported in acute HF- [14]and stable CAD patients.[29] Despite equal triglyceride loading of VLDL particles, NICM patients had 16% more VLDL particles than ICM patients. This aligns with a previous study reporting a higher atherogenic risk associated with 9% more VLDL particles in NICM compared to ICM patients.[10] VLDL fractions might be more atherogenic than LDL fractions, indicate a higher atherogenic burden in the NICM group.[30].

Several factors, such as HF severity, liver function, underlying CAD, statin therapy,[14] metabolic disorders, diet, and VLDL-HDL interactions, might mediate VLDL triglyceride metabolism differently between HF-etiologies.[31] The enzymes lipoprotein lipase and hepatic lipase facilitate triglyceride hydrolysis and VLDL clearance.[31], [32] Thus, enzyme availability or affinity, hepatic triglyceride lipidation, and/or metabolic de-lipidation of VLDLs might impact circulating VLDL levels in HF.[31] VLDL triglyceride content was unassociated with 1-year mortality in acute HF-patients,[14] possibly indicating less causality in HF outcomes compared to in atherogenic disease.[13], [30], [33], [34].

Higher cholesterol content in the large and medium-sized VLDL 1–4 subfractions in NICM patients aligns with a recent study,[10] indicating a higher atherogenic remnant cholesterol burden in NICM.[36] VLDL cholesterol loading might be a significant phenotype, as more severe HF was associated with fewer VLDL particles and lower cholesterol content. [10] Additionally, low VLDL cholesterol was linked to increased 1-year mortality in acute HF-patients,[14] while higher VLDL cholesterol load indicates less severe HF.[10] Reduced VLDL cholesterol in the sickest heart failure patients may result from hypoperfusion and congestion, negatively impacting hepatic perfusion, liver biosynthetic activity and nutrient absorption.[14] The complexity of VLDL subfractions and their prognostic importance should be further investigated to improve etiology-specific prevention and treatment of HF.[13], [34].

### 4.3 Intermediate-density lipoproteins

More IDL particles and higher triglycerides and cholesterol content in the NICM group compared to in the ICM group might indicate a higher atherogenic burden [26]. The atherogenic burden of remnant cholesterol, found in triglyceride-rich lipoproteins like VLDL and IDL, has been shown to increase the risk of peripheral artery disease, myocardial infarction, and ischemic stroke [36]. It also predicts coronary events and identify individuals who benefited most from statin therapy.[37] Progression of CAD has been more strongly related to IDL cholesterol content than circulating LDL fractions.[38] Therefor, ∼30% more IDL fractions with elevated cholesterol content might contribute to a higher atherogenic burden in NICM patients in our study. The prognostic importance of IDL fractions in HF should be further investigated.

### 4.4 Low-density lipoproteins

LDL showed the most systematic differences between etiologies, with twenty-nine LDL subfractions higher and more cholesterol and ApoB in LDL 1–6 subfractions in the NICM group. Similar discrepancies were recently reported in large, medium and small LDL particles [10]. Our NICM patients had LDL cholesterol levels (92.6 mg⋅dl^−1^) similar to healthy middle-aged participants (97.6 mg⋅dl^−1^, unpublished data), while levels in our ICM patients (63.5 mg⋅dl^−1^) were comparable to those in statin-treated CAD patients (68.4 mg⋅dl^-^) [25]. Both HF etiology groups had LDL cholesterol below the ∼102 and ∼118 mg⋅dl^−1^ reported in ischemic and non-ischemic HF patients in a recent study [10]. The discrepancies could be due to worse HF status, indicated by higher NT-proBNP levels and NYHA class in the previous study [10].

The LDL particle cholesterol content was similar in both groups (LDL cholesterol/ApoB, ∼1.3–1.4). However, the NICM group had a 26% higher atherogenic burden due to more LDL particles (LDL ApoB levels) [26]. A similar trend, with 14% more LDL particles in NICM compared to ICM patients, was previously reported [10]. Additionally, we found 16% more of the smallest and most atherogenic LDL 5–6 subfractions [39] in our NICM group, with LDL 5–6 contents comparable to levels previously reported in both CAD [25] and HF patients [10]. Beta-blocker treatment tends to increase small dense LDL subfractions [40]. Since most of our patients were on beta-blocker therapy [41], the etiology discrepancies are unlikely due to this medication. Others have suggested hepatic circulation, LDL production, LDL de-lipidation or LDL receptor activity as mechanisms behind etiology-specific lipid metabolism [10]. With similar inflammation, cachexia, triglycerides and heart failure severity biomarkers, statin treatment and etiology specific lipid metabolism might explain the observed etiology differences in our study.

### 4.5 High-density lipoproteins

Most HDL fractions and subfractions were similar across groups. Higher cholesterol in HDL 1 and HDL 3 in the NICM group suggests greater reversed cholesterol transport capacity, potentially modifying atherosclerosis [4], [42]. Low HDL levels are linked to CAD [43] and high triglycerides levels due to HDL − hepatic lipase interactions [33]. In HF, few small HDL particles and increased HDL cholesterol content are associated with adverse outcomes [9], [12]. Given minor etiology differences and low HDL subfraction heterogeneity, particularly in the small HDL 4 subfraction, HDL associated cardiovascular risk and the anti-atherogenic HDL activity was most likely similar across HF etiologies in our study.(4) The minor differences in HDL cholesterol content in our study might be due to older age in ICM patients rather than to HF etiology [44].

### 4.6 VLDL, LDL and left ventricular dimensions

LVEDV and LVEDD were weakly associated with triglyceride content in some VLDL and LDL subfractions, suggesting a link between these lipoproteins and LV size and function. Small post-prandial VLDL subfractions have been linked to left atrial remodeling in metabolic syndrome patients [45]. As VLDL is a major energy source for the heart, its subfractions might be association with LV dimensions and function [31]. Small, negatively charged VLDL subfractions may have higher cardiotoxicity [31], [45], potentially linking triglyceride-loaded VLDL subfractions to left ventricular dimensions in HFrEF patients. A UK biobank study found that higher LDL cholesterol and triglycerides were associated with increased LV mass and LVEDV [17]. While our study does not provide clear evidence, the LASSO model identified the variables most strongly associated with LVEDV and LVEDD in our patient populations. The clinical importance of these associations remains uncertain, and future research should explore underlying mechanisms, with studies in larger patient cohorts likely needed to further clarify these relationships.

## 5 Strengths and limitations

Our randomized multicenter study had high external validity, representing a heterogenic population of stable HFrEF patients. Using ^1^H NMR, we differentiated several lipoproteins, providing comprehensive reference values for VLDL, IDL, LDL and HDL fractions and sub-fractions. We found a strong correlation between biochemical and ^1^H NMR measurements.

However, our study has some limitations. The cross-sectional design limits the investigation of causal relationships. We did not measure key lipid enzymes like lipoprotein lipase, the LDL receptor, or the content of Lipoprotein (LP) (a). The absence of nutritional registrations and body composition measurement, which could affect lipid particle composition, is a limitation. We also did not differentiate between ApoB-100 and ApoB-48 chylomicron-associated particles or ApoA particles beyond ApoA-1 and ApoA-2. Detailed records of lipid-lowering treatment, including statins and combination therapies such as resins, fibrates, and omega-3 medication, were not maintained, as statin use was recorded only as a binary variable at inclusion. Given the timeline, potent lipid-lowering medications like Ezetimibe and PCSK9-inhibitors were likely not used by our patients. A potential etiology difference in statin therapy type or adherence remains unaccounted for, since we collected only self-reported statin use and not dosage and therapeutic goal attainment.

When statistically comparing several lipid subfraction, multiple testing may increase the likelihood of detecting differences by chance. Because we observed a consistent pattern across groups, and provided data variability using interquartile range, we did not apply correction for multiple statistical testing.

## 6 Conclusion

HF patients with reduced ejection fraction and non-ischemic etiology had the highest atherosclerotic lipoprotein burden due to more circulating ApoB carrying subfractions that were partly explained by less statin treatment. The triglyceride content of some VLDL and LDL subfractions was weakly associated with left ventricular structure and function. In conclusion, the findings indicate a role of considering lipoprotein subfractions for future prevention and treatment of HF, but the clinical importance must be evaluated in future studies.

## 7 Clinical implications

Our findings highlight HF etiology-specific lipid heterogeneity [10], potentially linked to liver hypoperfusion and congestion [14]. Recognizing and treating hypercholesterolemia and hypertriglyceridemia in non-ischemic heart failure patients seems crucial for personalized HFrEF care. Our results may serve as reference values for stable ischemic and non-ischemic HFrEF patients and inspire future research. Future studies should explore the link between lipid subfraction distribution and the severity of congestive heart failure, cachexia, or hepatic circulation.

## 8 Lay summary

We analyzed blood samples from heart failure patients to compare lipoproteins, which transport fatty acids and cholesterol, between two groups of heart failure patients. Our main findings were:

• Heart failure patients without established coronary artery disease had higher levels of harmful lipoproteins.
• This was partly due to fewer patients in this group taking lipid-lowering medication compared to those with established coronary artery disease and ischemic cardiomyopathy.

## 9 Declaration of generative ai and ai-assisted technologies in the manuscript preparation process

During the preparation of this work the first author used Microsoft Copilot for written English language improvements. After using this tool, the author reviewed and edited the content as needed and take full responsibility for the content of the published article.

## Authors contributions

TK, PB, CD, TH, AIL, AS, EVC, AL, EP, MH, ØE and HD made substantial contributions to the concept and design, acquisition of data, analysis and interpretation of data. TFB, TB and EKV made substantial contributions to analysis and interpretation of data. All authors in addition critically revised the article, gave final approval of the paper for publication and agreed to be accountable for all aspects of the work in ensuring that questions related to the accuracy and integrity of any part of the work are appropriately investigated.

## Data availability statement

The corresponding author can be contacted regarding data availability.

## CRediT authorship contribution statement

**Trine Karlsen:** Writing – review & editing, Writing – original draft, Project administration, Methodology, Investigation, Funding acquisition, Formal analysis, Data curation, Conceptualization. **Elisabeth K Vesterbekkmo:** Writing – review & editing, Writing – original draft, Methodology, Data curation, Conceptualization. **Torstein Hole:** Writing – review & editing, Project administration, Methodology, Investigation, Funding acquisition, Data curation, Conceptualization. **Alf Inge Larsen:** Writing – review & editing, Project administration, Methodology, Investigation, Funding acquisition, Formal analysis, Data curation, Conceptualization. **Torstein Valborgland:** Writing – review & editing, Resources, Project administration, Methodology, Investigation, Data curation. **Tonje Braaten:** Writing – review & editing, Validation, Supervision, Methodology, Formal analysis, Data curation. **Tone F Bathen:** Writing – review & editing, Resources, Methodology, Investigation, Data curation. **Paul Beckers:** Writing – review & editing, Project administration, Methodology, Investigation, Funding acquisition, Formal analysis, Data curation, Conceptualization. **Charles Delagardelle:** Writing – review & editing, Resources, Project administration, Methodology, Investigation, Funding acquisition, Formal analysis, Data curation, Conceptualization. **Patrick Feiereisen:** Writing – review & editing, Resources, Project administration, Methodology, Investigation, Funding acquisition, Formal analysis, Data curation, Conceptualization. **Emeline Van Craenenbroeck:** Writing – review & editing, Supervision, Resources, Project administration, Methodology, Investigation, Funding acquisition, Formal analysis, Data curation, Conceptualization. **Axel Linke:** Writing – review & editing, Supervision, Resources, Project administration, Methodology, Investigation, Funding acquisition, Formal analysis, Data curation, Conceptualization. **Eva Prescott:** Writing – review & editing, Supervision, Resources, Project administration, Methodology, Investigation, Funding acquisition, Formal analysis, Data curation, Conceptualization. **Martin Halle:** Writing – review & editing, Supervision, Resources, Project administration, Methodology, Investigation, Funding acquisition, Formal analysis, Data curation, Conceptualization. **Øyvind Ellingsen:** Writing – review & editing, Supervision, Resources, Project administration, Methodology, Investigation, Funding acquisition, Formal analysis, Data curation, Conceptualization. **Håvard Dalen:** Writing – review & editing, Supervision, Resources, Project administration, Methodology, Investigation, Funding acquisition, Formal analysis, Data curation, Conceptualization.

## Funding

St. Olavs Hospital; Faculty of Medicine, Norwegian University of Science and Technology; Norwegian Health Association; Danish Research Council; Central Norwegian Health Authority/Norwegian University of Science and Technology; Else-Kröner-Fresenius-Stiftung, and Société Luxembourgeoise pour la recherche sur les maladies cardiovasculaires.

## Declaration of competing interest

The authors declare the following financial interests/personal relationships which may be considered as potential competing interests: Trine Karlsen reports financial support was provided by Central Norwegian Health Authority. FUNDING St. Olavs Hospital; Faculty of Medicine, Norwegian University of Science and Technology; Norwegian Health Association; Danish Research Council; Central Norwegian Health Authority/Norwegian University of Science and Technology; Else-Kröner-Fresenius-Stiftung, and Société Luxembourgeoise pour la recherche sur les maladies cardiovasculaires. CONFLICTS OF INTEREST Dr Halle: member of the advisory board of Novartis, Sanofi-Aventis, and Merck Sharp & Dohme Corp, all outside of the present study. Dr Linke: Grants and personal fees from Medtronic and Claret Medical, as well as personal fees from Edwards, St. Jude Medical, Bard, and Symetis, all outside of the present study. Dr Dalen: Previous member of advisory board for Bristol Myers Squibb and Bohringer Ingelheim, both outside of the present study. Dr. Vesterbekkmo: Previous member of advisory board for Sanofi-Aventis and Novartis, all outside the present study.
