# Supplementary material for: Heart failure etiology and lipoprotein subfractions: Insight from the SMARTEX-HF study
Source: Int J Cardiol Heart Vasc. 2026 Feb 16;63:101888. doi: 10.1016/j.ijcha.2026.101888 (PMC12926980; doi:10.1016/j.ijcha.2026.101888)
Supplement: Supplementary Data 1 [file mmc1.docx]

Supplementary Table 1. Associations between heart failure etiology and lipid fractions and subfractions

| **Total serum** | Beta constant | R^2^ | F | p-value |
| --- | --- | --- | --- | --- |
| Triglycerides | 104.7 | 0.025 | 5.18 | 0.024 |
| Cholesterol | 114.8 | 0.156 | 37.46 | ˂0.001 |
| Free-cholesterol | 42.8 | 0.143 | 33.92 | ˂0.001 |
| ApoA-1 | 114.5 | 0.027 | 5.54 | 0.020 |
| ApoA-2 | 22.8 | 0.026 | 5.31 | 0.22 |
| ApoB | 55.4 | 0.130 | 30.33 | ˂0.001 |
| **VLDL** |  |  |  |  |
| Triglycerides | 68.1 | 0.018 | 3.65 | 0.058 |
| Cholesterol | 15.6 | 0.044 | 9.27 | 0.003 |
| Free-cholesterol | 8.8 | 0.035 | 7.44 | 0.007 |
| Phospholipids | 18.7 | 0.022 | 4.60 | 0.033 |
| ApoB | 8.0 | 0.031 | 6.48 | 0.012 |
| **IDL** |  |  |  |  |
| Triglyceride | 8.1 | 0.017 | 3.44 | 0.065 |
| Cholesterol | 2.5 | 0.091 | 20.26 | ˂0.001 |
| Free-cholesterol | 0.5 | 0.093 | 20.76 | ˂ 0.001 |
| Phospholipids | 1.6 | 0.048 | 10.27 | 0.002 |
| ApoB | 1.5 | 0.115 | 26.33 | ˂0.001 |
| **LDL** |  |  |  |  |
| Triglycerides | 13.7 | 0.091 | 20.26 | ˂0.001 |
| Cholesterol | 51.3 | 0.087 | 19.38 | ˂0.001 |
| Free-cholesterol | 19.5 | 0.069 | 15.04 | ˂0.001 |
| Phospholipids | 33.8 | 0.084 | 18.52 | ˂0.001 |
| ApoB | 41.0 | 0.101 | 22.78 | ˂0.001 |
| **HDL** |  |  |  |  |
| Triglycerides | 8.5 | 0.018 | 3.81 | 0.052 |
| Cholesterol | 42.6 | 0.012 | 2.52 | 0.114 |
| Free-cholesterol | 12.4 | 0.015 | 3.09 | 0.080 |
| Phospholipids | 58.2 | 0.010 | 1.96 | 0.163 |
| ApoA-1 | 117.1 | 0.016 | 3.23 | 0.074 |
| ApoA-2 | 24.0 | 0.027 | 5.63 | 0.019 |
|  |  |  |  |  |
| VLDL 1 cholesterol | 5.5 | 0.031 | 6.42 | 0.012 |
| VLDL 2 cholesterol | 2.3 | 0.037 | 7.78 | 0.006 |
| VLDL 3 cholesterol | 1.6 | 0.045 | 9.56 | 0.002 |
| VLDL 4 cholesterol | 3.0 | 0.062 | 13.46 | ˂0.001 |
| VLDL 5 cholesterol | 2.0 | 0.000 | 0.01 | 0.906 |
| VLDL 6 cholesterol | 0.0 | 0.015 | 3.07 | 0.081 |
|  |  |  |  |  |
| VLDL 1 triglycerides | 34.1 | 0.013 | 2,74 | 0.099 |
| VLDL 2 triglycerides | 12.4 | 0.008 | 1.61 | 0.206 |
| VLDL 3 triglycerides | 9.3 | 0.019 | 4.01 | 0.046 |
| VLDL 4 triglycerides | 7.8 | 0.030 | 6.35 | 0.013 |
| VLDL 5 triglycerides | 4.0 | 0.012 | 2.51 | ˂ 0.001 |
| VLDL 6 triglycerides | 0.2 | 0.074 | 16.30 | ˂0.001 |
|  |  |  |  |  |
| VLDL 1 free-cholesterol | 1.3 | 0.016 | 3.37 | 0.068 |
| VLDL 2 free-cholesterol | 0.9 | 0.037 | 7.77 | 0.006 |
| VLDL 3 free-cholesterol | 1.0 | 0.030 | 6.35 | 0.013 |
| VLDL 4 free-cholesterol | 1.0 | 0.069 | 15.07 | ˂0.001 |
| VLDL 5 free-cholesterol | 0.9 | 0.047 | 9.99 | 0.002 |
| VLDL 6 free-cholesterol | 0.1 | 0.030 | 6.34 | 0.013 |
|  |  |  |  |  |
| VLDL 1 phospholipids | 4.9 | 0.011 | 2.18 | 0.142 |
| VLDL 2 phospholipids | 2.6 | 0.010 | 2.15 | 0.144 |
| VLDL 3 phospholipids | 2.2 | 0.026 | 5.33 | 0.022 |
| VLDL 4 phospholipids | 3.5 | 0.049 | 10.50 | 0.001 |
| VLDL 5 phospholipids | 2.5 | 0.002 | 0.14 | 0.550 |
| VLDL 6 phospholipids | 0.2 | 0.031 | 6.46 | 0.012 |
|  |  |  |  |  |
| LDL 1 cholesterol | 8.0 | 0.136 | 31.84 | ˂0.001 |
| LDL 2 cholesterol | 4.8 | 0.075 | 16.36 | ˂0.001 |
| LDL 3 cholesterol | 3.5 | 0.057 | 12.25 | ˂0.001 |
| LDL 4 cholesterol | 1.7 | 0.034 | 7.22 | 0.008 |
| LDL 5 cholesterol | 10.1 | 0.026 | 5.52 | 0.020 |
| LDL 6 cholesterol | 17.3 | 0.051 | 10.868 | 0.001 |
|  |  |  |  |  |
| LDL 1 Triglycerides | 4.4 | 0.080 | 17.604 | ˂0.001 |
| LDL 2 Triglycerides | 0.9 | 0.124 | 28.755 | ˂0.001 |
| LDL 3 Triglycerides | 2.1 | 0.086 | 19.17 | ˂0.001 |
| LDL 4 Triglycerides | 1.7 | 0.034 | 7.219 | 0.008 |
| LDL 5 Triglycerides | 1.9 | 0.054 | 11.504 | ˂0.001 |
| LDL 6 Triglycerides | 4.1 | 0.020 | 4.198 | 0.042 |
|  |  |  |  |  |
| LDL 1 free-cholesterol | 2.6 | 0.136 | 31.887 | ˂0.001 |
| LDL 2 free-cholesterol | 2.3 | 0.080 | 17.55 | ˂0.001 |
| LDL 3 free-cholesterol | 2.7 | 0.046 | 9.84 | 0.002 |
| LDL 4 free-cholesterol | 2.7 | 0.021 | 4.45 | 0.036 |
| LDL 5 free-cholesterol | 3.4 | 0.021 | 4.34 | 0.038 |
| LDL 6 free-cholesterol | 4.3 | 0.026 | 5.39 | 0.021 |
|  |  |  |  |  |
| LDL 1 Phospholipids | 6.0 | 0.129 | 30.08 | ˂0.001 |
| LDL 2 Phospholipids | 3.4 | 0.075 | 16.40 | ˂0.001 |
| LDL 3 Phospholipids | 2.8 | 0.050 | 10.74 | 0.001 |
| LDL 4 Phospholipids | 3.9 | 0.028 | 5.76 | 0.017 |
| LDL 5 Phospholipids | 6.1 | 0.022 | 4.59 | 0.033 |
| LDL 6 Phospholipids | 9.9 | 0.048 | 10.15 | 0.002 |
|  |  |  |  |  |
| LDL 1 ApoB | 5.9 | 0.130 | 30.40 | ˂0.001 |
| LDL 2 ApoB | 3.8 | 0.075 | 16.40 | ˂0.001 |
| LDL 3 ApoB | 3.0 | 0.054 | 11.57 | ˂0.001 |
| LDL 4 ApoB | 4.2 | 0.033 | 7.01 | 0.009 |
| LDL 5 ApoB | 7.6 | 0.027 | 5.73 | 0.018 |
| LDL 6 ApoB | 14.6 | 0.053 | 11.41 | ˂0.001 |
|  |  |  |  |  |
| HDL 1 cholesterol | 8.7 | 0.025 | 5.28 | 0.023 |
| HDL 2 cholesterol | 5.0 | 0.024 | 5.07 | 0.025 |
| HDL 3 cholesterol | 7.3 | 0.016 | 3.22 | 0.074 |
| HDL 4 cholesterol | 19.0 | 0.002 | 0.33 | 0.564 |
|  |  |  |  |  |
| HDL 1 triglycerides | 1.9 | 0.023 | 4.79 | 0.030 |
| HDL 2 triglycerides | 1.3 | 0.012 | 2.57 | 0.111 |
| HDL 3 triglycerides | 1.8 | 0.012 | 2.49 | 0.116 |
| HDL 4 triglycerides | 3.6 | 0.008 | 1.62 | 0.204 |
|  |  |  |  |  |
| HDL 1 free-cholesterol | 2.4 | 0.025 | 5.28 | 0.023 |
| HDL 2 free-cholesterol | 2.9 | 0.011 | 2.22 | 0.138 |
| HDL 3 free-cholesterol | 1.5 | 0.005 | 1.09 | 0.298 |
| HDL 4 free-cholesterol | 3.8 | 0.000 | 0.08 | 0.777 |
|  |  |  |  |  |
| HDL 1 phospholipids | 10.9 | 0.017 | 3.49 | 0.063 |
| HDL 2 phospholipids | 8.1 | 0.021 | 4.36 | 0.038 |
| HDL 3 phospholipids | 11.8 | 0.008 | 1.66 | 0.199 |
| HDL 4 phospholipids | 25.7 | 0.002 | 0.31 | 0.581 |
|  |  |  |  |  |
| HDL 1 ApoA-1 | 13.6 | 0.015 | 3.05 | 0.082 |
| HDL 2 ApoA-1 | 13.1 | 0.024 | 4.95 | 0.027 |
| HDL 3 ApoA-1 | 21.1 | 0.026 | 5.48 | 0.020 |
| HDL 4 ApoA-1 | 72.3 | 0.000 | 0.05 | 0.826 |
|  |  |  |  |  |
| HDL 1 ApoA-2 | 0.7 | 0.038 | 7.94 | 0.005 |
| HDL 2 ApoA-2 | 1.1 | 0.068 | 14.78 | ˂0.001 |
| HDL 3 ApoA-2 | 3.7 | 0.044 | 9.45 | 0.002 |
| HDL 4 ApoA-2 | 17.0 | 0.000 | 0.08 | 0.782 |

ApoB = apolipoprotein B; ApoA-1 = apolipoprotein A-1; ApoA-2 = apolipoprotein A-2; VLDL = very-low-density lipoprotein fraction; IDL = intermediate-density lipoprotein fraction; LDL = low-density lipoprotein fraction; HDL = high-density lipoprotein fraction

Supplementary Table 2. Associations between heart failure etiology, statin treatment and lipid fractions and subfractions

| **Total serum** | Beta constant | R^2^ | F | p-value |
| --- | --- | --- | --- | --- |
| Triglyceride | 32.4 | 0.044 | 4.67 | 0.010 |
| Cholesterol | 168.3 | 0.237 | 31.45 | ˂0.001 |
| Free-cholesterol | 59.0 | 0.235 | 31.10 | ˂0.001 |
| ApoA-1 | 112.5 | 0.017 | 2.81 | 0.062 |
| ApoA-2 | 21.2 | 0.030 | 3.15 | 0.045 |
| ApoB | 85.4 | 0.225 | 29.34 | ˂0.001 |
| **VLDL** |  |  |  |  |
| Triglycerides | 11.5 | 0.041 | 4.30 | 0.015 |
| Cholesterol | 10.0 | 0.050 | 5.32 | 0.006 |
| Free-cholesterol | 5.2 | 0.050 | 5.36 | 0.005 |
| Phospholipids | 10.0 | 0.041 | 4.28 | 0.015 |
| ApoB | 5.5 | 0.042 | 4.44 | 0.013 |
| **IDL** |  |  |  |  |
| Triglyceride | -3.1 | 0.032 | 3.37 | 0.036 |
| Cholesterol | 3.2 | 0.091 | 10.12 | ˂0.001 |
| Free-cholesterol | 0.4 | 0.093 | 10.33 | ˂ 0.001 |
| Phospholipids | 0.6 | 0.050 | 5.29 | 0.006 |
| ApoB | 1.3 | 0.115 | 13.14 | ˂0.001 |
| **LDL** |  |  |  |  |
| Triglycerides | 19.0 | 0.120 | 13.84 | ˂0.001 |
| Cholesterol | 108.5 | 0.256 | 34.67 | ˂0.001 |
| Free-cholesterol | 35.8 | 0.247 | 33.05 | ˂0.001 |
| Phospholipids | 63.7 | 0.266 | 36.57 | ˂0.001 |
| ApoB | 76.4 | 0.271 | 37.56 | ˂0.001 |
| **HDL** |  |  |  |  |
| Triglycerides | 6.4 | 0.036 | 3.79 | 0.024 |
| Cholesterol | 43.5 | 0.013 | 1.30 | 0.275 |
| Free-cholesterol | 12.8 | 0.016 | 1.61 | 0.202 |
| Phospholipids | 58.9 | 0.010 | 0.99 | 0.372 |
| ApoA-1 | 115.4 | 0.016 | 1.66 | 0.196 |
| ApoA-2 | 22.6 | 0.031 | 3.25 | 0.041 |
|  |  |  |  |  |
| VLDL 1 cholesterol | -0.9 | 0.057 | 6.06 | 0.003 |
| VLDL 2 cholesterol | 2.2 | 0.037 | 3.87 | 0.022 |
| VLDL 3 cholesterol | 1.30 | 0.045 | 4.81 | 0.009 |
| VLDL 4 cholesterol | 3.5 | 0.064 | 6.91 | 0.001 |
| VLDL 5 cholesterol | 2.1 | 0.001 | 0.06 | 0.942 |
| VLDL 6 cholesterol | 0.0 | 0.026 | 2.71 | 0.069 |
|  |  |  |  |  |
| VLDL 1 triglycerides | -13.1 | 0.051 | 5.41 | 0.005 |
| VLDL 2 triglycerides | 8.9 | 0.011 | 1.14 | 0.322 |
| VLDL 3 triglycerides | 6.6 | 0.023 | 2.33 | 0.100 |
| VLDL 4 triglycerides | 5.3 | 0.039 | 4.15 | 0.017 |
| VLDL 5 triglycerides | 3.3 | 0.045 | 4.81 | 0.009 |
| VLDL 6 triglycerides | -0.1 | 0.080 | 8.81 | ˂0.001 |
|  |  |  |  |  |
| VLDL 1 free-cholesterol | -1.2 | 0.043 | 4.57 | 0.012 |
| VLDL 2 free-cholesterol | 0.5 | 0.041 | 4.36 | 0.014 |
| VLDL 3 free-cholesterol | 0.3 | 0.038 | 3.96 | 0.021 |
| VLDL 4 free-cholesterol | 0.7 | 0.072 | 7.78 | ˂0.001 |
| VLDL 5 free-cholesterol | 0.3 | 0.091 | 10.10 | ˂0.001 |
| VLDL 6 free-cholesterol | 0.1 | 0.038 | 3.97 | 0.020 |
|  |  |  |  |  |
| VLDL 1 phospholipids | -1.0 | 0.039 | 4.08 | 0.018 |
| VLDL 2 phospholipids | 2.0 | 0.012 | 1.27 | 0.283 |
| VLDL 3 phospholipids | 1.2 | 0.030 | 3.12 | 0.044 |
| VLDL 4 phospholipids | 3.0 | 0.052 | 5.55 | 0.05 |
| VLDL 5 phospholipids | 2.3 | 0.005 | 0.55 | 0.577 |
| VLDL 6 phospholipids | 0.2 | 0.039 | 4.07 | 0.019 |
|  |  |  |  |  |
| LDL 1 cholesterol | 17.7 | 0.229 | 29.97 | ˂0.001 |
| LDL 2 cholesterol | 12.9 | 0.159 | 19.06 | ˂0.001 |
| LDL 3 cholesterol | 14.2 | 0.198 | 24.98 | ˂0.001 |
| LDL 4 cholesterol | 3.3 | 0.155 | 18.49 | ˂0.001 |
| LDL 5 cholesterol | 17.8 | 0.087 | 9.59 | ˂0.001 |
| LDL 6 cholesterol | 18.6 | 0.052 | 5.521 | 0.005 |
|  |  |  |  |  |
| LDL 1 Triglycerides | 4.7 | 0.080 | 8.826 | ˂0.001 |
| LDL 2 Triglycerides | 1.8 | 0.179 | 22.052 | ˂0.001 |
| LDL 3 Triglycerides | 3.0 | 0.177 | 21.652 | ˂0.001 |
| LDL 4 Triglycerides | 3.3 | 0.155 | 18.487 | ˂0.001 |
| LDL 5 Triglycerides | 3.2 | 0.129 | 14.948 | ˂0.001 |
| LDL 6 Triglycerides | 3.0 | 0.020 | 2.111 | 0.124 |
|  |  |  |  |  |
| LDL 1 free-cholesterol | 5.6 | 0.238 | 31.520 | ˂0.001 |
| LDL 2 free-cholesterol | 4.2 | 0.129 | 14.90 | ˂0.001 |
| LDL 3 free-cholesterol | 5.8 | 0.179 | 21.95 | ˂0.001 |
| LDL 4 free-cholesterol | 5.2 | 0.107 | 12.14 | ˂0.001 |
| LDL 5 free-cholesterol | 5.6 | 0.098 | 10.93 | ˂0.001 |
| LDL 6 free-cholesterol | 5.1 | 0.032 | 3.32 | 0.038 |
|  |  |  |  |  |
| LDL 1 Phospholipids | 10.6 | 0.209 | 26.75 | ˂0.001 |
| LDL 2 Phospholipids | 7.8 | 0.170 | 20.74 | ˂0.001 |
| LDL 3 Phospholipids | 8.7 | 0.201 | 25.48 | ˂0.001 |
| LDL 4 Phospholipids | 8.7 | 0.109 | 12.38 | ˂0.001 |
| LDL 5 Phospholipids | 10.1 | 0.088 | 9.69 | ˂0.001 |
| LDL 6 Phospholipids | 10.3 | 0.048 | 5.08 | 0.007 |
|  |  |  |  |  |
| LDL 1 ApoB | 10.5 | 0.214 | 27.44 | ˂0.001 |
| LDL 2 ApoB | 8.3 | 0.175 | 21.46 | ˂0.001 |
| LDL 3 ApoB | 9.6 | 0.225 | 29.39 | ˂0.001 |
| LDL 4 ApoB | 10.8 | 0.120 | 13.78 | ˂0.001 |
| LDL 5 ApoB | 13.2 | 0.097 | 10.83 | ˂0.001 |
| LDL 6 ApoB | 14.2 | 0.053 | 5.69 | 0.004 |
|  |  |  |  |  |
| HDL 1 cholesterol | 9.1 | 0.026 | 2.65 | 0.073 |
| HDL 2 cholesterol | 4.5 | 0.027 | 2.81 | 0.063 |
| HDL 3 cholesterol | 6.9 | 0.017 | 1.78 | 0.172 |
| HDL 4 cholesterol | 19.7 | 0.003 | 0.31 | 0.735 |
|  |  |  |  |  |
| HDL 1 triglycerides | 0.9 | 0.036 | 3.79 | 0.024 |
| HDL 2 triglycerides | 0.8 | 0.031 | 3.24 | 0.041 |
| HDL 3 triglycerides | 1.3 | 0.032 | 3.29 | 0.039 |
| HDL 4 triglycerides | 3.1 | 0.023 | 2.39 | 0.094 |
|  |  |  |  |  |
| HDL 1 free-cholesterol | 2.9 | 0.030 | 3.09 | 0.048 |
| HDL 2 free-cholesterol | 1.3 | 0.014 | 1.43 | 0.241 |
| HDL 3 free-cholesterol | 1.9 | 0.021 | 2.17 | 0.117 |
| HDL 4 free-cholesterol | 4.5 | 0.013 | 1.37 | 0.257 |
|  |  |  |  |  |
| HDL 1 phospholipids | 11.1 | 0.017 | 1.74 | 0.178 |
| HDL 2 phospholipids | 7.1 | 0.026 | 2.67 | 0.072 |
| HDL 3 phospholipids | 11.5 | 0.009 | 0.87 | 0.422 |
| HDL 4 phospholipids | 26.8 | 0.004 | 0.36 | 0.696 |
|  |  |  |  |  |
| HDL 1 ApoA-1 | 13.2 | 0.015 | 1.53 | 0.220 |
| HDL 2 ApoA-1 | 11.3 | 0.034 | 3.53 | 0.031 |
| HDL 3 ApoA-1 | 18.2 | 0.041 | 4.33 | 0.014 |
| HDL 4 ApoA-1 | 74.9 | 0.002 | 0.23 | 0.799 |
|  |  |  |  |  |
| ^b)^ HDL 1 ApoA-2 | 0.6 | 0.038 | 3.99 | 0.020 |
| ^b)^ HDL 2 ApoA-2 | 0.6 | 0.076 | 8.36 | ˂0.001 |
| ^b)^ HDL 3 ApoA-2 | 3.3 | 0.049 | 5.19 | 0.006 |
| ^b)^ HDL 4 ApoA-2 | 17.1 | 0.000 | 0.04 | 0.961 |

ApoB = apolipoprotein B; ApoA-1 = apolipoprotein A-1; ApoA-2 = apolipoprotein A-2; VLDL = very-low-density lipoprotein fraction; IDL = intermediate-density lipoprotein fraction; LDL = low-density lipoprotein fraction; HDL = high-density lipoprotein fraction.

Supplementary Table 3. Association between heart failure etiology, statin treatment, age, and lipid fractions and subfractions.

| **Total serum** | Beta constant | R^2^ | F | p-value |
| --- | --- | --- | --- | --- |
| Triglyceride | 145.6 | 0.073 | 5.28 | 0.002 |
| Cholesterol | 194.0 | 0.249 | 22.21 | ˂0.001 |
| Free-cholesterol | 61.9 | 0.237 | 20.84 | ˂0.001 |
| ApoA-1 | 113.4 | 0.027 | 1.87 | 0.136 |
| ApoA-2 | 27.4 | 0.076 | 5.48 | 0.001 |
| ApoB | 97.2 | 0.234 | 20.49 | ˂0.001 |
| **VLDL** |  |  |  |  |
| Triglycerides | 94.0 | 0.071 | 5.11 | 0.002 |
| Cholesterol | 27.2 | 0.087 | 6.41 | ˂0.001 |
| Free-cholesterol | 11.4 | 0.078 | 5.67 | ˂0.001 |
| Phospholipids | 24.4 | 0.071 | 5.14 | 0.002 |
| ApoB | 9.8 | 0.063 | 4.53 | 0.004 |
| **IDL** |  |  |  |  |
| Triglyceride | 17.8 | 0.066 | 4.70 | 0.003 |
| Cholesterol | 11.8 | 0.123 | 9.43 | ˂0.001 |
| Free-cholesterol | 3.0 | 0.127 | 9.74 | ˂ 0.001 |
| Phospholipids | 6.7 | 0.088 | 6.45 | ˂0.001 |
| ApoB | 3.4 | 0.134 | 10.35 | ˂0.001 |
| **LDL** |  |  |  |  |
| Triglycerides | 20.5 | 0.122 | 9.32 | ˂0.001 |
| Cholesterol | 115.3 | 0.257 | 23.18 | ˂0.001 |
| Free-cholesterol | 34.7 | 0.247 | 21.99 | ˂0.001 |
| Phospholipids | 65.1 | 0.266 | 24.29 | ˂0.001 |
| ApoB | 82.4 | 0.274 | 25.29 | ˂0.001 |
| **HDL** |  |  |  |  |
| Triglycerides | 6.2 | 0.036 | 2.52 | 0.059 |
| Cholesterol | 39.6 | 0.017 | 1.18 | 0.320 |
| Free-cholesterol | 9.4 | 0.047 | 3.31 | 0.021 |
| Phospholipids | 53.3 | 0.015 | 1.04 | 0.378 |
| ApoA-1 | 114.2 | 0.016 | 1.10 | 0.352 |
| ApoA-2 | 29.0 | 0.082 | 6.00 | ˂0.001 |
|  |  |  |  |  |
| VLDL 1 cholesterol | 9.5 | 0.098 | 7.28 | ˂0.001 |
| VLDL 2 cholesterol | 5.5 | 0.079 | 5.77 | ˂0.001 |
| VLDL 3 cholesterol | 4.2 | 0.074 | 5.38 | 0.001 |
| VLDL 4 cholesterol | 4.7 | 0.071 | 5.09 | 0.002 |
| VLDL 5 cholesterol | 1.9 | 0.002 | 0.17 | 0.918 |
| VLDL 6 cholesterol | 0.0 | 0.028 | 1.91 | 0.129 |
|  |  |  |  |  |
| VLDL 1 triglycerides | 43.2 | 0.083 | 6.09 | ˂0.001 |
| VLDL 2 triglycerides | 21.0 | 0.036 | 2.49 | 0.062 |
| VLDL 3 triglycerides | 15.4 | 0.043 | 3.02 | 0.031 |
| VLDL 4 triglycerides | 8.1 | 0.047 | 3.28 | 0.022 |
| VLDL 5 triglycerides | 3.1 | 0.049 | 3.46 | 0.017 |
| VLDL 6 triglycerides | 0.6 | 0.097 | 7.22 | ˂0.001 |
|  |  |  |  |  |
| VLDL 1 free-cholesterol | 2.6 | 0.081 | 5.90 | ˂0.001 |
| VLDL 2 free-cholesterol | 2.1 | 0.074 | 5.37 | 0.001 |
| VLDL 3 free-cholesterol | 2.0 | 0.069 | 4.95 | 0.002 |
| VLDL 4 free-cholesterol | 1.4 | 0.079 | 5.78 | ˂0.001 |
| VLDL 5 free-cholesterol | 0.8 | 0.104 | 7.75 | ˂0.001 |
| VLDL 6 free-cholesterol | 0.1 | 0.075 | 5.43 | 0.001 |
|  |  |  |  |  |
| VLDL 1 phospholipids | 8.1 | 0.080 | 5.81 | ˂0.001 |
| VLDL 2 phospholipids | 5.0 | 0.044 | 3.06 | 0.029 |
| VLDL 3 phospholipids | 4.0 | 0.057 | 4.01 | 0.008 |
| VLDL 4 phospholipids | 4.1 | 0.060 | 4.29 | 0.006 |
| VLDL 5 phospholipids | 2.2 | 0.006 | 0.42 | 0.736 |
| VLDL 6 phospholipids | 0.3 | 0.075 | 5.48 | 0.001 |
|  |  |  |  |  |
| LDL 1 cholesterol | 16.7 | 0.229 | 19.95 | ˂0.001 |
| LDL 2 cholesterol | 13.9 | 0.159 | 12.71 | ˂0.001 |
| LDL 3 cholesterol | 12.1 | 0.202 | 16.91 | ˂0.001 |
| LDL 4 cholesterol | 12.4 | 0.102 | 8.72 | ˂0.001 |
| LDL 5 cholesterol | 21.0 | 0.093 | 6.87 | ˂0.001 |
| LDL 6 cholesterol | 27.2 | 0.076 | 5.532 | 0.001 |
|  |  |  |  |  |
| LDL 1 Triglycerides | 5.7 | 0.084 | 6.126 | ˂0.001 |
| LDL 2 Triglycerides | 1.4 | 0.186 | 15.344 | ˂0.001 |
| LDL 3 Triglycerides | 2.4 | 0.199 | 16.611 | ˂0.001 |
| LDL 4 Triglycerides | 2.3 | 0.180 | 14.721 | ˂0.001 |
| LDL 5 Triglycerides | 3.7 | 0.134 | 10.336 | ˂0.001 |
| LDL 6 Triglycerides | 5.3 | 0.033 | 2.259 | 0.083 |
|  |  |  |  |  |
| LDL 1 free-cholesterol | 5.4 | 0.238 | 20.949 | ˂0.001 |
| LDL 2 free-cholesterol | 4.5 | 0.129 | 9.95 | ˂0.001 |
| LDL 3 free-cholesterol | 4.9 | 0.186 | 15.29 | ˂0.001 |
| LDL 4 free-cholesterol | 4.1 | 0.118 | 8.99 | ˂0.001 |
| LDL 5 free-cholesterol | 5.9 | 0.098 | 7.32 | ˂0.001 |
| LDL 6 free-cholesterol | 6.8 | 0.051 | 3.66 | 0.014 |
|  |  |  |  |  |
| LDL 1 Phospholipids | 9.7 | 0.211 | 17.95 | ˂0.001 |
| LDL 2 Phospholipids | 7.9 | 0.170 | 13.74 | ˂0.001 |
| LDL 3 Phospholipids | 7.2 | 0.207 | 17.53 | ˂0.001 |
| LDL 4 Phospholipids | 6.8 | 0.117 | 8.85 | ˂0.001 |
| LDL 5 Phospholipids | 11.6 | 0.093 | 6.87 | ˂0.001 |
| LDL 6 Phospholipids | 14.8 | 0.074 | 5.32 | 0.002 |
|  |  |  |  |  |
| LDL 1 ApoB | 9.3 | 0.217 | 18.58 | ˂0.001 |
| LDL 2 ApoB | 8.6 | 0.176 | 14.27 | ˂0.001 |
| LDL 3 ApoB | 8.0 | 0.231 | 20.18 | ˂0.001 |
| LDL 4 ApoB | 8.4 | 0.124 | 9.51 | ˂0.001 |
| LDL 5 ApoB | 15.7 | 0.105 | 7.82 | ˂0.001 |
| LDL 6 ApoB | 22.8 | 0.080 | 5.85 | ˂0.001 |
|  |  |  |  |  |
| HDL 1 cholesterol | 3.7 | 0.051 | 3.57 | 0.015 |
| HDL 2 cholesterol | 3.1 | 0.041 | 2.87 | 0.038 |
| HDL 3 cholesterol | 7.3 | 0.018 | 1.22 | 0.302 |
| HDL 4 cholesterol | 23.0 | 0.020 | 1.35 | 0.260 |
|  |  |  |  |  |
| HDL 1 triglycerides | -0.1 | 0.044 | 3.09 | 0.028 |
| HDL 2 triglycerides | 0.6 | 0.034 | 2.33 | 0.075 |
| HDL 3 triglycerides | 1.8 | 0.042 | 2.95 | 0.034 |
| HDL 4 triglycerides | 4.3 | 0.074 | 5.32 | 0.002 |
|  |  |  |  |  |
| HDL 1 free-cholesterol | 1.5 | 0.051 | 3.60 | 0.015 |
| HDL 2 free-cholesterol | 1.0 | 0.021 | 1.47 | 0.224 |
| HDL 3 free-cholesterol | 2.0 | 0.021 | 1.44 | 0.231 |
| HDL 4 free-cholesterol | 4.6 | 0.014 | 0.92 | 0.431 |
|  |  |  |  |  |
| HDL 1 phospholipids | 3.0 | 0.059 | 4.19 | 0.007 |
| HDL 2 phospholipids | 5.0 | 0.041 | 2.83 | 0.040 |
| HDL 3 phospholipids | 12.4 | 0.011 | 0.73 | 0.538 |
| HDL 4 phospholipids | 31.2 | 0.023 | 1.58 | 0.197 |
|  |  |  |  |  |
| HDL 1 ApoA-1 | 2.0 | 0.055 | 3.91 | 0.010 |
| HDL 2 ApoA-1 | 10.6 | 0.035 | 2.40 | 0.069 |
| HDL 3 ApoA-1 | 19.5 | 0.043 | 3.00 | 0.032 |
| HDL 4 ApoA-1 | 84.6 | 0.018 | 1.26 | 0.290 |
|  |  |  |  |  |
| HDL 1 ApoA-2 | 0.0 | 0.046 | 3.21 | 0.024 |
| HDL 2 ApoA-2 | 0.9 | 0.079 | 5.77 | ˂0.001 |
| HDL 3 ApoA-2 | 4.7 | 0.078 | 5.67 | ˂0.001 |
| HDL 4 ApoA-2 | 22.8 | 0.071 | 5.10 | 0.002 |

ApoB = apolipoprotein B; ApoA-1 = apolipoprotein A1; ApoA-2 = apolipoprotein A2; VLDL = very-low-density lipoprotein fraction; IDL = intermediate-density lipoprotein fraction; LDL = low-density lipoprotein fraction; HDL = high-density lipoprotein fraction.

Supplementary table 4. Association between heart failure etiology, statin treatment, age, body mass index, and lipid fractions and subfractions

| **Total serum** | Beta constant | R^2^ | F | p-value |
| --- | --- | --- | --- | --- |
| Triglyceride | -41.7 | 0.146 | 8.57 | ˂0.001 |
| Cholesterol | 190.4 | 0.249 | 16.59 | ˂0.001 |
| Free-cholesterol | 62.7 | 0.237 | 15.56 | ˂0.001 |
| ApoA-1 | 130.4 | 0.048 | 2.52 | 0.042 |
| ApoA-2 | 29.3 | 0.080 | 4.32 | 0.002 |
| **VLDL** |  |  |  |  |
| Triglycerides | -44.1 | 0.149 | 8.76 | ˂0.001 |
| Cholesterol | 3.7 | 0.151 | 8.92 | ˂0.001 |
| Free-cholesterol | 0.9 | 0.151 | 8.89 | ˂0.001 |
| Phospholipids | -0.1 | 0.153 | 9.07 | ˂0.001 |
| ApoB | 1.2 | 0.142 | 8.27 | ˂0.001 |
| **IDL** |  |  |  |  |
| Triglyceride | -14.1 | 0.138 | 7.97 | ˂0.001 |
| Cholesterol | 0.6 | 0.173 | 10.48 | ˂0.001 |
| Free-cholesterol | -0.1 | 0.173 | 10.49 | ˂ 0.001 |
| Phospholipids | -1.3 | 0.148 | 8.70 | ˂0.001 |
| ApoB | -0.1 | 0.181 | 11.05 | ˂0.001 |
| **LDL** |  |  |  |  |
| Triglycerides | 14.5 | 0.145 | 8.45 | ˂0.001 |
| Cholesterol | 130.4 | 0.264 | 17.90 | ˂0.001 |
| Free-cholesterol | 42.5 | 0.270 | 18.50 | ˂0.001 |
| Phospholipids | 74.6 | 0.276 | 19.10 | ˂0.001 |
| ApoB | 83.3 | 0.274 | 18.88 | ˂0.001 |
| **HDL** |  |  |  |  |
| Triglycerides | 3.3 | 0.056 | 2.96 | 0.021 |
| Cholesterol | 50.0 | 0.048 | 2.50 | 0.044 |
| Free-cholesterol | 14.9 | 0.123 | 6.99 | ˂0.001 |
| Phospholipids | 70.0 | 0.055 | 2.92 | 0.022 |
| ApoA-1 | 134.5 | 0.043 | 2.22 | 0.068 |
| ApoA-2 | 29.7 | 0.083 | 4.51 | 0.002 |
|  |  |  |  |  |
| VLDL 1 cholesterol | -5.6 | 0.178 | 10.84 | ˂0.001 |
| VLDL 2 cholesterol | 1.9 | 0.126 | 7.23 | ˂0.001 |
| VLDL 3 cholesterol | 1.0 | 0.107 | 5.98 | ˂0.001 |
| VLDL 4 cholesterol | 2.9 | 0.084 | 4.60 | 0.001 |
| VLDL 5 cholesterol | 1.9 | 0.003 | 0.13 | 0.973 |
| VLDL 6 cholesterol | 0.0 | 0.082 | 4.49 | 0.002 |
|  |  |  |  |  |
| VLDL 1 triglycerides | -51.7 | 0.169 | 10.18 | ˂0.001 |
| VLDL 2 triglycerides | 1.9 | 0.093 | 5.15 | ˂0.001 |
| VLDL 3 triglycerides | 2.2 | 0.086 | 4.70 | 0.001 |
| VLDL 4 triglycerides | 0.5 | 0.097 | 5.38 | ˂0.001 |
| VLDL 5 triglycerides | 2.2 | 0.084 | 4.59 | 0.001 |
| VLDL 6 triglycerides | -0.3 | 0.123 | 7.00 | ˂0.001 |
| VLDL 1 free-cholesterol | -3.4 | 0.171 | 10.29 | ˂0.001 |
| VLDL 2 free-cholesterol | -0.1 | 0.130 | 7.45 | ˂0.001 |
| VLDL 3 free-cholesterol | -0.4 | 0.128 | 7.33 | ˂0.001 |
| VLDL 4 free-cholesterol | 0.2 | 0.103 | 5.76 | ˂0.001 |
| VLDL 5 free-cholesterol | -0.0 | 0.140 | 8.12 | ˂0.001 |
| VLDL 6 free-cholesterol | 0.1 | 0.145 | 8.46 | ˂0.001 |
|  |  |  |  |  |
| VLDL 1 phospholipids | -5.8 | 0.170 | 10.21 | ˂0.001 |
| VLDL 2 phospholipids | 0.7 | 0.104 | 5.79 | ˂0.001 |
| VLDL 3 phospholipids | -0.1 | 0.110 | 6.19 | ˂0.001 |
| VLDL 4 phospholipids | 1.5 | 0.098 | 5.41 | ˂0.001 |
| VLDL 5 phospholipids | 2.0 | 0.010 | 0.49 | 0.740 |
| VLDL 6 phospholipids | 0.2 | 0.145 | 8.48 | ˂0.001 |
|  |  |  |  |  |
| LDL 1 cholesterol | 20.0 | 0.235 | 15.39 | ˂0.001 |
| LDL 2 cholesterol | 15.5 | 0.161 | 9.62 | ˂0.001 |
| LDL 3 cholesterol | 19.1 | 0.235 | 15.40 | ˂0.001 |
| LDL 4 cholesterol | 19.7 | 0.145 | 8.46 | ˂0.001 |
| LDL 5 cholesterol | 22.2 | 0.094 | 5.17 | ˂0.001 |
| LDL 6 cholesterol | 15.0 | 0.122 | 6.966 | ˂0.001 |
|  |  |  |  |  |
| LDL 1 Triglycerides | 2.1 | 0.127 | 7.271 | ˂0.001 |
| LDL 2 Triglycerides | 1.3 | 0.187 | 11.530 | ˂0.001 |
| LDL 3 Triglycerides | 2.6 | 0.201 | 12.589 | ˂0.001 |
| LDL 4 Triglycerides | 3.3 | 0.203 | 12.759 | ˂0.001 |
| LDL 5 Triglycerides | 3.3 | 0.137 | 7.962 | ˂0.001 |
| LDL 6 Triglycerides | 2.3 | 0.086 | 4.734 | 0.001 |
|  |  |  |  |  |
| LDL 1 free-cholesterol | 6.3 | 0.244 | 16.137 | ˂0.001 |
| LDL 2 free-cholesterol | 5.2 | 0.132 | 7.63 | ˂0.001 |
| LDL 3 free-cholesterol | 7.4 | 0.236 | 15.43 | ˂0.001 |
| LDL 4 free-cholesterol | 6.3 | 0.156 | 9.23 | ˂0.001 |
| LDL 5 free-cholesterol | 6.8 | 0.105 | 5.87 | ˂0.001 |
| LDL 6 free-cholesterol | 4.9 | 0.073 | 3.96 | 0.004 |
|  |  |  |  |  |
| LDL 1 Phospholipids | 11.1 | 0.216 | 13.75 | ˂0.001 |
| LDL 2 Phospholipids | 9.2 | 0.175 | 10.64 | ˂0.001 |
| LDL 3 Phospholipids | 11.1 | 0.247 | 16.37 | ˂0.001 |
| LDL 4 Phospholipids | 11.0 | 0.151 | 8.89 | ˂0.001 |
| LDL 5 Phospholipids | 12.4 | 0.094 | 5.20 | ˂0.001 |
| LDL 6 Phospholipids | 8.6 | 0.119 | 6.73 | ˂0.001 |
|  |  |  |  |  |
| LDL 1 ApoB | 10.1 | 0.219 | 13.98 | ˂0.001 |
| LDL 2 ApoB | 9.3 | 0.177 | 10.75 | ˂0.001 |
| LDL 3 ApoB | 11.6 | 0.262 | 17.72 | ˂0.001 |
| LDL 4 ApoB | 12.9 | 0.153 | 9.03 | ˂0.001 |
| LDL 5 ApoB | 15.6 | 0.105 | 5.84 | ˂0.001 |
| LDL 6 ApoB | 10.5 | 0.132 | 7.60 | ˂0.001 |
| HDL 1 cholesterol | 5.1 | 0.052 | 2.75 | 0.029 |
| HDL 2 cholesterol | 4.7 | 0.059 | 3.12 | 0.016 |
| HDL 3 cholesterol | 9.3 | 0.044 | 2.33 | 0.058 |
| HDL 4 cholesterol | 26.8 | 0.041 | 2.15 | 0.076 |
|  |  |  |  |  |
| HDL 1 triglycerides | -1.5 | 0.057 | 3.02 | 0.019 |
| HDL 2 triglycerides | 0.0 | 0.053 | 2.81 | 0.027 |
| HDL 3 triglycerides | 0.9 | 0.082 | 4.49 | 0.002 |
| HDL 4 triglycerides | 3.3 | 0.107 | 6.00 | ˂0.001 |
|  |  |  |  |  |
| HDL 1 free-cholesterol | 3.6 | 0.100 | 5.56 | ˂0.001 |
| HDL 2 free-cholesterol | 2.0 | 0.090 | 4.95 | ˂0.001 |
| HDL 3 free-cholesterol | 2.9 | 0.081 | 4.39 | 0.002 |
| HDL 4 free-cholesterol | 6.3 | 0.068 | 3.67 | 0.007 |
|  |  |  |  |  |
| HDL 1 phospholipids | 7.4 | 0.070 | 3.78 | 0.005 |
| HDL 2 phospholipids | 7.2 | 0.055 | 2.90 | 0.023 |
| HDL 3 phospholipids | 14.8 | 0.025 | 1.29 | 0.277 |
| HDL 4 phospholipids | 36.1 | 0.046 | 2.40 | 0.051 |
|  |  |  |  |  |
| HDL 1 ApoA-1 | 8.9 | 0.069 | 3.72 | 0.006 |
| HDL 2 ApoA-1 | 12.9 | 0.044 | 2.29 | 0.061 |
| HDL 3 ApoA-1 | 22.1 | 0.050 | 2.62 | 0.036 |
| HDL 4 ApoA-1 | 94.7 | 0.035 | 1.82 | 0.126 |
|  |  |  |  |  |
| HDL 1 ApoA-2 | 0.5 | 0.050 | 2.64 | 0.035 |
| HDL 2 ApoA-2 | 0.6 | 0.082 | 4.47 | 0.002 |
| HDL 3 ApoA-2 | 4.5 | 0.078 | 4.26 | 0.003 |
| HDL 4 ApoA-2 | 24.2 | 0.075 | 4.05 | 0.004 |

ApoB = apolipoprotein B; ApoA-1 = apolipoprotein A-1; ApoA-2 = apolipoprotein A-2; VLDL = very-low-density lipoprotein fraction; IDL = intermediate-density lipoprotein fraction; LDL = low-density lipoprotein fraction; HDL = high-density lipoprotein fraction.

Supplementary Table 5. Associations between heart failure etiology, statin treatment, peak oxygen uptake, and lipid fractions and subfractions

| **Total serum** | Beta constant | R^2^ | F | p-value |
| --- | --- | --- | --- | --- |
| Triglyceride | 0.6 | 0.050 | 3.50 | 0.016 |
| Cholesterol | 151.1 | 0.250 | 22.32 | ˂0.001 |
| Free-cholesterol | 55.5 | 0.242 | 21.37 | ˂0.001 |
| ApoA-1 | 106.4 | 0.034 | 2.36 | 0.073 |
| ApoA-2 | 18.5 | 0.051 | 3.58 | 0.015 |
| ApoB | 80.2 | 0.229 | 19.94 | ˂0.001 |
| **VLDL** |  |  |  |  |
| Triglycerides | -10.9 | 0.046 | 3.24 | 0.023 |
| Cholesterol | 6.3 | 0.054 | 3.83 | 0.110 |
| Free-cholesterol | 3.5 | 0.055 | 3.92 | 0.010 |
| Phospholipids | 6.6 | 0.045 | 3.14 | 0.026 |
| ApoB | 4.8 | 0.043 | 3.03 | 0.030 |
| **IDL** |  |  |  |  |
| Triglyceride | -8.8 | 0.038 | 2.68 | 0.048 |
| Cholesterol | 2.1 | 0.092 | 6.80 | ˂0.001 |
| Free-cholesterol | 0.0 | 0.095 | 7.03 | ˂0.001 |
| Phospholipids | -0.9 | 0.055 | 3.90 | 0.010 |
| ApoB | 1.3 | 0.115 | 8.71 | ˂0.001 |
| **LDL** |  |  |  |  |
| Triglycerides | 20.4 | 0.124 | 9.48 | ˂0.001 |
| Cholesterol | 99.1 | 0.262 | 23.82 | ˂0.001 |
| Free-cholesterol | 34.4 | 0.249 | 22.17 | ˂0.001 |
| Phospholipids | 60.0 | 0.270 | 24.77 | ˂0.001 |
| ApoB | 72.2 | 0.275 | 25.36 | ˂0.001 |
| **HDL** |  |  |  |  |
| Triglycerides | 7.2 | 0.039 | 2.76 | 0.044 |
| Cholesterol | 39.6 | 0.024 | 1.63 | 0.185 |
| Free-cholesterol | 11.7 | 0.023 | 1.56 | 0.196 |
| Phospholipids | 55.6 | 0.014 | 0.98 | 0.404 |
| ApoA-1 | 108.3 | 0.024 | 1.67 | 0.176 |
| ApoA-2 | 20.1 | 0.050 | 3.54 | 0.016 |
|  |  |  |  |  |
| VLDL 1 cholesterol | -3.8 | 0.064 | 4.58 | 0.004 |
| VLDL 2 cholesterol | 1.6 | 0.041 | 2.86 | 0.038 |
| VLDL 3 cholesterol | 0.8 | 0.048 | 3.36 | 0.020 |
| VLDL 4 cholesterol | 3.7 | 0.065 | 4.6 | 0.004 |
| VLDL 5 cholesterol | 2.0 | 0.002 | 0.14 | 0.939 |
| VLDL 6 cholesterol | 0.0 | 0.034 | 2.36 | 0.073 |
|  |  |  |  |  |
| VLDL 1 triglycerides | -30.1 | 0.058 | 4.12 | 0.007 |
| VLDL 2 triglycerides | 6.1 | 0.014 | 0.98 | 0.406 |
| VLDL 3 triglycerides | 4.3 | 0.026 | 1.78 | 0.153 |
| VLDL 4 triglycerides | 4.9 | 0.040 | 2.78 | 0.042 |
| VLDL 5 triglycerides | 3.3 | 0.046 | 3.23 | 0.024 |
| VLDL 6 triglycerides | 0.1 | 0.082 | 6.00 | ˂0.001 |
|  |  |  |  |  |
| VLDL 1 free-cholesterol | -2.1 | 0.049 | 3.49 | 0.017 |
| VLDL 2 free-cholesterol | 0.1 | 0.046 | 3.21 | 0.024 |
| VLDL 3 free-cholesterol | -0.1 | 0.041 | 2.88 | 0.037 |
| VLDL 4 free-cholesterol | 0.7 | 0.072 | 5.16 | 0.002 |
| VLDL 5 free-cholesterol | 0.1 | 0.097 | 7.20 | ˂0.001 |
| VLDL 6 free-cholesterol | 0.1 | 0.044 | 3.06 | 0.029 |
|  |  |  |  |  |
| VLDL 1 phospholipids | -3.3 | 0.045 | 3.17 | 0.026 |
| VLDL 2 phospholipids | 1.5 | 0.015 | 1.03 | 0.380 |
| VLDL 3 phospholipids | 0.8 | 0.032 | 2.24 | 0.085 |
| VLDL 4 phospholipids | 3.0 | 0.052 | 3.68 | 0.013 |
| VLDL 5 phospholipids | 2.2 | 0.010 | 0.67 | 0.571 |
| VLDL 6 phospholipids | 0.2 | 0.045 | 3.13 | 0.027 |
|  |  |  |  |  |
| LDL 1 cholesterol | 17.6 | 0.229 | 19.88 | ˂0.001 |
| LDL 2 cholesterol | 10.1 | 0.173 | 14.06 | ˂0.001 |
| LDL 3 cholesterol | 12.3 | 0.205 | 17.25 | ˂0.001 |
| LDL 4 cholesterol | 13.0 | 0.118 | 8.93 | ˂0.001 |
| LDL 5 cholesterol | 16.4 | 0.090 | 6.62 | ˂0.001 |
| LDL 6 cholesterol | 15.7 | 0.059 | 4.173 | 0.007 |
|  |  |  |  |  |
| LDL 1 Triglycerides | 5.0 | 0.081 | 5.924 | ˂0.001 |
| LDL 2 Triglycerides | 2.1 | 0.185 | 15.221 | ˂0.001 |
| LDL 3 Triglycerides | 3.2 | 0.182 | 14.931 | ˂0.001 |
| LDL 4 Triglycerides | 3.8 | 0.170 | 13.693 | ˂0.001 |
| LDL 5 Triglycerides | 3.5 | 0.134 | 10.390 | ˂0.001 |
| LDL 6 Triglycerides | 3.5 | 0.023 | 1.579 | 0.196 |
|  |  |  |  |  |
| LDL 1 free-cholesterol | 5.7 | 0.238 | 20.941 | ˂0.001 |
| LDL 2 free-cholesterol | 3.3 | 0.143 | 11.17 | ˂0.001 |
| LDL 3 free-cholesterol | 5.4 | 0.182 | 14.93 | ˂0.001 |
| LDL 4 free-cholesterol | 4.9 | 0.110 | 8.26 | ˂0.001 |
| LDL 5 free-cholesterol | 5.4 | 0.099 | 7.33 | ˂0.001 |
| LDL 6 free-cholesterol | 4.4 | 0.038 | 2.65 | 0.050 |
|  |  |  |  |  |
| LDL 1 Phospholipids | 10.9 | 0.210 | 17.78 | ˂0.001 |
| LDL 2 Phospholipids | 6.5 | 0.182 | 14.95 | ˂0.001 |
| LDL 3 Phospholipids | 7.9 | 0.206 | 17.35 | ˂0.001 |
| LDL 4 Phospholipids | 7.8 | 0.113 | 8.53 | ˂0.001 |
| LDL 5 Phospholipids | 9.5 | 0.090 | 6.61 | ˂0.001 |
|  |  |  |  |  |
| LDL 6 Phospholipids | 8.6 | 0.056 | 3.97 | 0.009 |
| LDL 1 ApoB | 10.9 | 0.215 | 18.31 | ˂0.001 |
| LDL 2 ApoB | 7.1 | 0.186 | 15.27 | ˂0.001 |
| LDL 3 ApoB | 9.0 | 0.227 | 19.70 | ˂0.001 |
| LDL 4 ApoB | 9.3 | 0.122 | 9.33 | ˂0.001 |
| LDL 5 ApoB | 12.8 | 0.097 | 7.24 | ˂0.001 |
| LDL 6 ApoB | 11.3 | 0.061 | 4.34 | 0.005 |
|  |  |  |  |  |
| HDL 1 cholesterol | 9.3 | 0.026 | 1.77 | 0.155 |
| HDL 2 cholesterol | 3.9 | 0.033 | 2.31 | 0.078 |
| HDL 3 cholesterol | 5.9 | 0.035 | 2.44 | 0.065 |
| HDL 4 cholesterol | 17.4 | 0.024 | 1.61 | 0.187 |
|  |  |  |  |  |
| HDL 1 triglycerides | 1.4 | 0.041 | 2.89 | 0.037 |
| HDL 2 triglycerides | 1.0 | 0.036 | 2.47 | 0.063 |
| HDL 3 triglycerides | 1.3 | 0.032 | 2.18 | 0.091 |
| HDL 4 triglycerides | 3.0 | 0.025 | 1.75 | 0.157 |
|  |  |  |  |  |
| HDL 1 free-cholesterol | 2.6 | 0.031 | 2.15 | 0.095 |
| HDL 2 free-cholesterol | 1.2 | 0.015 | 1.05 | 0.372 |
| HDL 3 free-cholesterol | 1.7 | 0.029 | 2.03 | 0.112 |
| HDL 4 free-cholesterol | 4.0 | 0.023 | 1.54 | 0.205 |
|  |  |  |  |  |
| HDL 1 phospholipids | 12.2 | 0.019 | 1.29 | 0.278 |
| HDL 2 phospholipids | 6.6 | 0.028 | 1.94 | 0.124 |
| HDL 3 phospholipids | 10.2 | 0.021 | 1.40 | 0.243 |
| HDL 4 phospholipids | 24.0 | 0.023 | 1.55 | 0.202 |
|  |  |  |  |  |
| HDL 1 ApoA-1 | 15.0 | 0.017 | 1.19 | 0.316 |
| HDL 2 ApoA-1 | 10.5 | 0.037 | 2.56 | 0.056 |
| HDL 3 ApoA-1 | 15.5 | 0.060 | 4.26 | 0.006 |
| HDL 4 ApoA-1 | 69.2 | 0.016 | 1.09 | 0.355 |
|  |  |  |  |  |
| HDL 1 ApoA-2 | 0.7 | 0.038 | 2.65 | 0.050 |
| HDL 2 ApoA-2 | 0.3 | 0.079 | 5.76 | ˂0.001 |
| HDL 3 ApoA-2 | 2.6 | 0.063 | 4.48 | 0.005 |
| HDL 4 ApoA-2 | 15.0 | 0.023 | 1.60 | 0.191 |

ApoB = apolipoprotein B; ApoA-1 = apolipoprotein A-1; ApoA-2 = apolipoprotein A-2; VLDL = very-low-density lipoprotein fraction; IDL = intermediate-density lipoprotein fraction; LDL = low-density lipoprotein fraction; HDL = high-density lipoprotein fraction.
