# Supplementary material for: Heart failure etiology and lipoprotein subfractions: Insight from the SMARTEX-HF study
Source: Int J Cardiol Heart Vasc. 2026 Feb 16;63:101888. doi: 10.1016/j.ijcha.2026.101888 (PMC12926980; doi:10.1016/j.ijcha.2026.101888)
Supplement: Supplementary Data 2 [file mmc2.docx]

Supplementary Table 6. Mixed effects regression analysis (LASSO)

|  |  | Coefficient | 95% CI | P value |
| --- | --- | --- | --- | --- |
| LVEDV | | | | |
|  | VLDL 5 triglycerides | -15.32 | -29.29 - -1.34 | 0.032 |
|  | LDL 2 triglycerides | -18.77 | -33.28 - 4.25 | 0.011 |
|  | LDL 5 triglycerides | 4.94 | -10.12 – 20.00 | 0.520 |
|  | LDL 5 ApoB | -0.31 | -3.21 – 2.58 | 0.833 |
|  | HDL 4 triglycerides | 0.23 | -12.77 – 13.23 | 0.972 |
|  | IDL free cholesterol | 5.95 | -1.00-12.90 | 0.094 |
| LVEDD | | | | |
|  | VLDL 4 triglycerides | -0.21 | -0.47 – 0.05 | 0.113 |
|  | VLDL 5 triglycerides | -0.21 | -1.87 – 1.45 | 0.803 |
|  | VLDL 6 triglycerides | 1.75 | 0.15 – 3.35 | 0.032 |
|  | LDL 2 triglycerides | -1.44 | -2.96 – 0.08 | 0.064 |
|  | HDL 1 cholesterol | -0.16 | -0.36 – 0.03 | 0.106 |
| LVEF | | | | |
|  | VLDL 4 triglycerides | 0.08 | -0.09 – 0.26 | 0.351 |
|  | LDL 2 triglycerides | 0.64 | -0.54 – 1.83 | 0.289 |
| S’ | | | | |
|  | VLDL 5 triglycerides | 0.24 | 0.03 – 0.45 | 0.024 |

CI = confidence interval; LVEDV = Left ventricular end-diastolic volume; VLDL = very-low-density lipoprotein; LDL = low-density lipoprotein; APOB = Apolipoprotein B; HDL = high-density lipoprotein; IDL = intermediate density lipoprotein; LVEDD = left ventricular end-diastolic diameter; LVEF = Left ventricular ejection fraction; S’ = Mitral annular systolic peak velocity.
